# Rheumatoid arthritis synovial fluid induces JAK-dependent intracellular activation of human sensory neurons

**DOI:** 10.1172/jci.insight.186646

**Published:** 2025-05-15

**Authors:** Yuening Li, Elizabeth H. Gray, Rosie Ross, Irene Zebochin, Amy Lock, Laura Fedele, Louisa Janice Kamajaya, Rebecca J. Marrow, Sarah Ryan, Pascal Röderer, Oliver Brüstle, Susan John, Franziska Denk, Leonie S. Taams

**Affiliations:** 1 Centre for Inflammation Biology & Cancer Immunology, Department of Inflammation Biology, School of Immunology & Microbial Sciences,; 2 Wolfson Sensory, Pain and Regeneration Centre (SPaRC), Guy’s Campus, and; 3 Peter Gorer Department of Immunobiology, School of Immunology & Microbial Sciences, King’s College London, London, United Kingdom.; 4 Institute of Reconstructive Neurobiology, University of Bonn Medical Faculty & University Hospital Bonn, Bonn, Germany.; 5 LIFE&BRAIN GmbH, Cellomics Unit, Bonn, Germany.

**Keywords:** Immunology, Inflammation, Neuroscience, Pain, Rheumatology, iPS cells

## Abstract

JAK inhibitors (JAKi) are widely used antiinflammatory drugs. Recent data suggest that JAKi have superior effects on pain reduction in rheumatoid arthritis (RA). However, the underlying mechanisms for this observation are not fully understood. We investigated whether JAKi can act directly on human sensory neurons. We analyzed RNA-seq datasets of sensory neurons and found that they expressed JAK1 and STAT3. Addition of cell-free RA synovial fluid to human induced pluripotent stem cell–derived (iPSC-derived) sensory neurons led to phosphorylation of STAT3 (pSTAT3), which was completely blocked by the JAKi tofacitinib. Compared with paired serum, RA synovial fluid was enriched for the STAT3 signalling cytokines IL-6, IL-11, LIF, IFN-α, and IFN-β, with their requisite receptors present in peripheral nerves postmortem. Accordingly, these recombinant cytokines induced pSTAT3 in iPSC-derived sensory neurons. Furthermore, IL-6 + sIL-6R and LIF upregulated expression of pain-relevant genes with STAT3-binding sites, an effect that was blocked by tofacitinib. LIF also induced neuronal sensitization, highlighting this molecule as a putative pain mediator. Finally, over time, tofacitinib reduced the firing rate of sensory neurons stimulated with RA synovial fluid. Together, these data indicate that JAKi can act directly on human sensory neurons, providing a potential mechanistic explanation for their suggested superior analgesic properties.

## Abstract 2

This manuscript provides evidence for a direct effect of JAK inhibitors on sensory neurons – potentially explaining why these compounds have superior analgesic action in arthritis.

## Graphical abstract

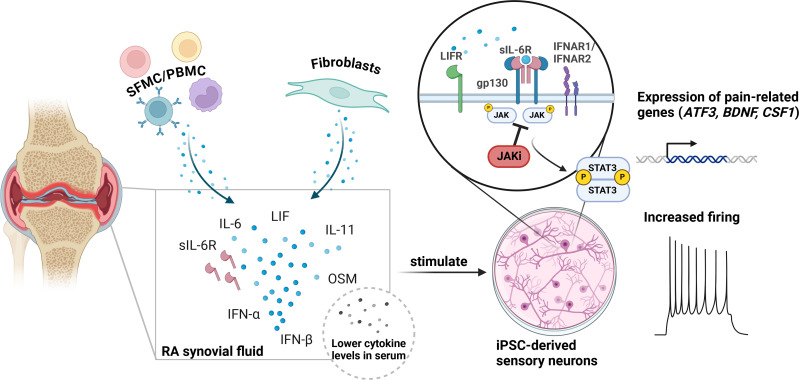

## Introduction

Rheumatoid arthritis (RA) is a chronic autoimmune disease characterized by inflammation, stiffness, and bilateral joint pain (1, 2). The advent of biologics has revolutionized the treatment of RA, leading to substantial reduction in overall disease activity. However, moderate to severe pain persists in many patients, despite improved treatments, and remains a critical unmet need for individuals living with RA (3, 4).

Among current treatments, a class of small-molecule drugs that inhibit Janus kinase (JAK) proteins, called JAK inhibitors (JAKi), show promising results in terms of pain relief. JAKi work by inhibiting the ATP binding domains of JAK, disabling cytokine binding–induced phosphorylation and its downstream effects (5). Multiple cytokines signal through JAK proteins and their partner, Signal Transducer and Activator of Transcription (STAT), molecules. JAKi can block multiple cytokines simultaneously, which may underlie their high efficacy (6). Several lines of evidence suggest that JAKi may be superior to other treatments, including anti-TNF, in reducing pain in RA. Head-to-head studies showed that the clinically approved JAKi upadacitinib and baricitinib performed better on secondary outcome measures such as worst joint pain and mean visual analogue scale for pain (7, 8). Post-hoc mediator analyses also suggested that baricitinib may directly inhibit pain, as pain reduction exceeded the reduction in inflammatory markers in individuals with RA (9).

The mechanisms behind the proposed superior analgesic efficacy of JAKi remain unclear. Pain in RA is initiated by the activation of sensory neurons that innervate inflamed joints and transmit noxious information to the central nervous system, where the pain percept is generated (10). Many mechanistic studies of JAKi have focused on their inhibition of immune cell activation and proliferation (5, 11), which would indirectly reduce pain. It has also been suggested that JAKi might be able to act directly on pain pathways in the central nervous system (12). Here, we explored the possibility that the same might be true for peripheral sensory neurons — the first responders that become aberrantly activated in pain states. Specifically, we hypothesized that JAK/STAT signalling cytokines in the inflamed RA joint exert direct effects on sensory neuron activation, which can be blocked by JAKi.

We tested this hypothesis using induced pluripotent stem cell–derived (iPSC-derived) sensory neurons as a model system. Our findings demonstrate that JAK signalling is active in sensory neurons and can be driven by multiple cytokines present in the joint fluid of individuals with RA. We propose this direct action on sensory neurons as one putative explanation for the suggested superior analgesic efficacy of JAKi.

## Results

**Figure 1:**
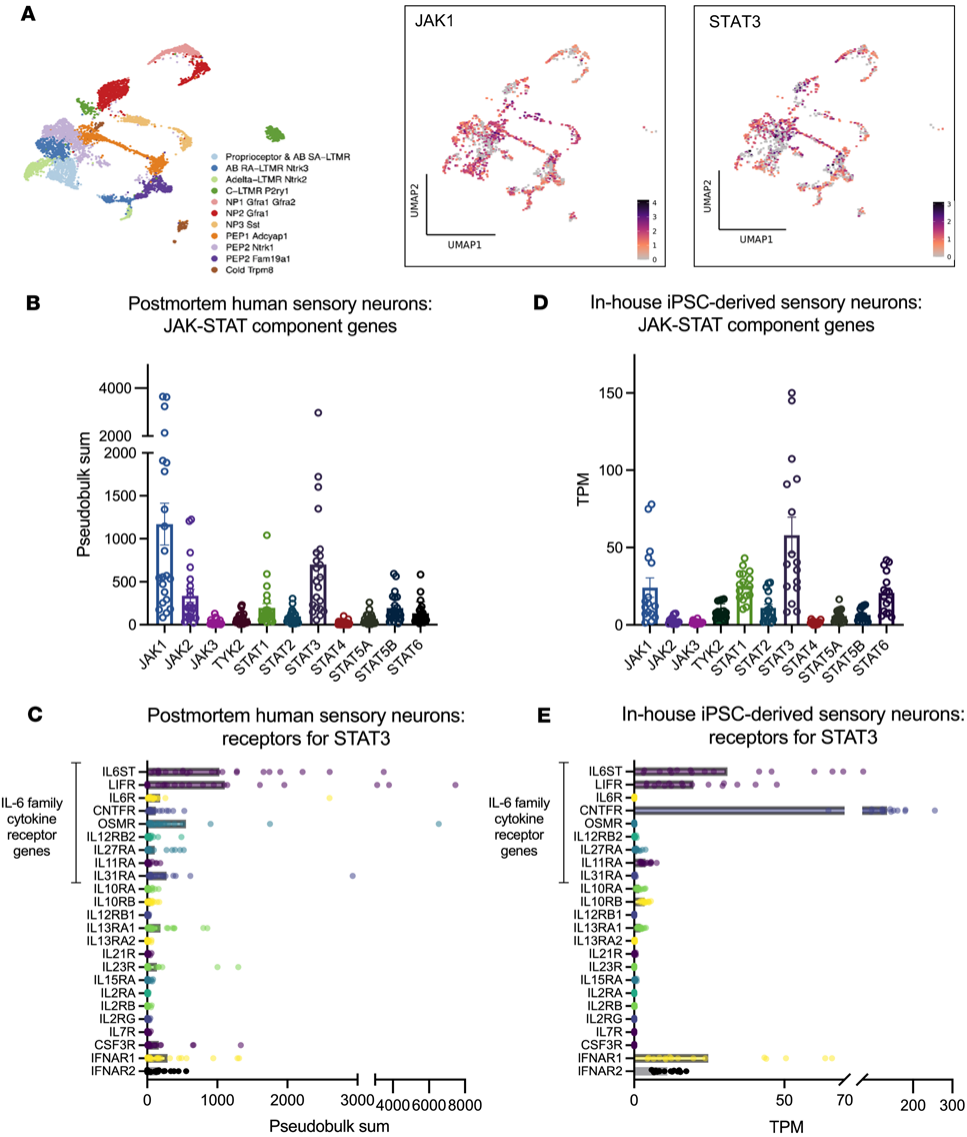
Genes related to JAK/STAT signalling are expressed in human sensory neurons and iPSC-derived sensory neurons. (**A**) Single-nuclei RNA-seq datasets (XSpecies DRG Atlas: http://research-pub.gene.com/XSpeciesDRGAtlas/) (13) were analyzed for the expression of JAK1 and STAT3 across different subtypes of human postmortem sensory neurons. Color scales indicate scaled average expression across samples. (**B**–**E**) Pseudobulk analyses of single-nuclei RNA-seq of human postmortem (13, 14) (**B**) and in-house iPSC-derived sensory neurons for the indicated JAK and STAT molecules (17) (**D**). Expression patterns of the receptors for the indicated STAT3 signalling cytokines in human postmortem (**C**) and in-house iPSC-derived sensory neurons (**E**). TPM, transcripts per million. Error bars in **B** and **D** indicate SEM.

We first interrogated publicly available databases for the expression levels of the components of JAK/STAT signalling in human sensory neurons. Analyses of two human postmortem dorsal root ganglia (DRG) neuron single-nuclei RNA-seq studies and one compilation study showed that JAK1 and STAT3 are widely expressed in human sensory neurons, at levels higher than other JAKs and STATs (13–15) (Figure 1, A and B, and Supplemental Figure 1A; supplemental material available online with this article; https://doi.org/10.1172/jci.insight.186646DS1). We investigated which STAT3-signalling cytokines may be able to induce neuronal pSTAT3 directly, by identifying the transcript levels of their corresponding receptors on postmortem human sensory neurons (Figure 1C). We found that *IL6ST*, *LIFR*, *OSMR*, *IL31RA*, *IFNAR1*, and *IFNAR2* were moderately to highly expressed, highlighting a possible role for IL-6 family cytokines and type-I IFNs in sensory neuron activation.

**Figure 2:**
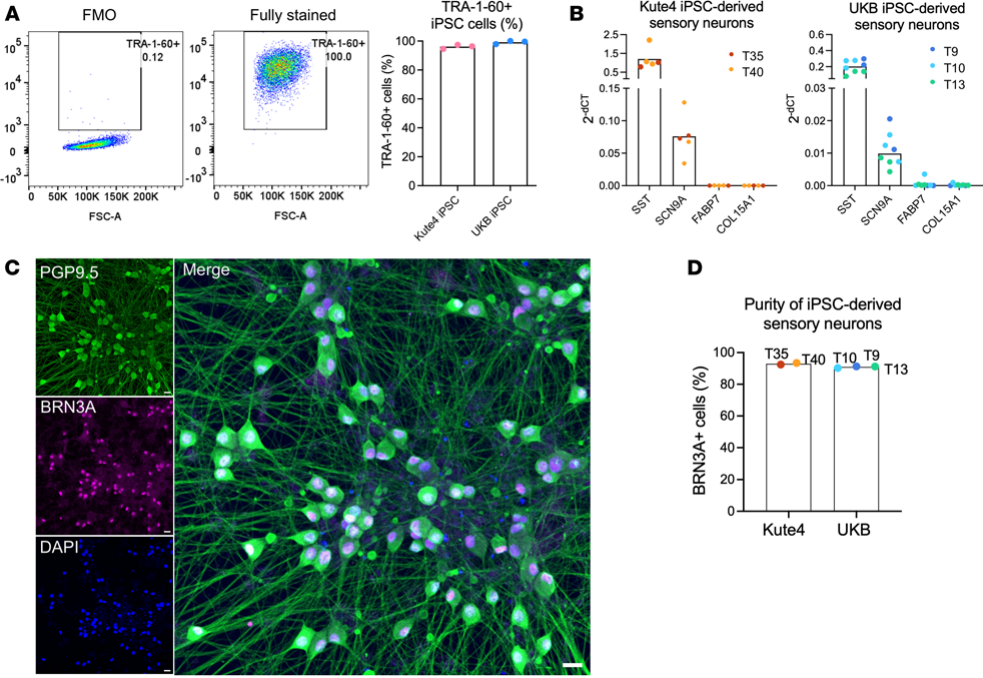
Characterization of iPSC-derived sensory neurons. (**A**) IPSC-derived sensory neurons were differentiated from Kute4 and UKB lines and stained for the expression of TRA-1-60, a marker of undifferentiated cells. Representative flow cytometry plots and cumulative data (*n* = 3) showing the percentage of TRA-1-60+ cells in UKB iPSC at passage 14, as compared to the fluorescence minus one (FMO) control (left panel). (**B**) Bar plots showing gene expression of neuronal markers (*SST*, *SCN9A*) and nonneuronal genes (*FABP7*, *COL15A1*) by sensory neurons derived from Kute4 (left) and UKB (right) iPSC lines. Within each independent differentiation batch (indicated by trial (T) number and different colors) *n* = 2–3 biological repeats were used. (**C** and **D**) IPSC-derived sensory neurons were differentiated to over day 50 and stained for the sensory neuron marker BRN3A and panneuronal marker PGP9.5. Representative IHC of day 81 neuron staining shown in **C** (Scale bar: 20 μm) and cumulative data showing the purity of the neuronal culture, quantified as BRN3A^+^ cells within DAPI+ cells (**D**, *n* = 5 independent differentiation batches of Kute4 and UKB iPSC lines).

As a next step, we generated human iPSC-derived sensory neurons to use as a model system. These cells are less heterogenous than native human nociceptors, usually differentiating into a single, homogenous population of sensory neurons that is phenotypically “mixed,” i.e., expresses a range of mediators that are not always coexpressed in a single neuron subtype in vivo (16). Crucially for the present study, however, in-house sequencing data showed that our iPSC-derived sensory neurons appeared to recapitulate native JAK/STAT gene expression (17) (Figure 1D). These results were in keeping with a publicly available atlas of sensory neurons differentiated with a similar protocol (18) (Supplemental Figure 1B). Moreover, with some exceptions (*IL31RA*, *OSMR*, and *CNTFR),* the expression patterns of JAK/STAT3–relevant cytokine receptors were largely similar to human postmortem sensory neurons. In particular, high abundance of *IL6ST*, *LIFR*, *IFNAR1*, and *IFNAR2* was recapitulated (Figure 1E and Supplemental Figure 1B). In order to investigate this further at a cellular and functional level, we derived sensory neurons from 2 iPSC lines, Kute4 and UKB. We first confirmed that both iPSC lines were in an undifferentiated state using flow cytometry for the marker TRA-1-60 (Figure 2A). Upon differentiation using the Chambers protocol (19), sensory neurons expressed neuronal markers somatostatin (*SST*) and Nav1.7 (*SCN9A*), with glial and fibroblast markers *FABP7* and *COL15A1* largely absent (Figure 2B). At the protein level, most of the neurons were positive for BRN3A, a sensory neuron transcription factor, and PGP9.5, a panneuronal marker, with a purity of over 90% across all the batches used in this study (Figure 2, C and D). These data indicate that our iPSC-derived sensory neurons were pure, with a phenotype similar to human sensory neurons, and therefore suitable for studying STAT3 signalling.

**Figure 3:**
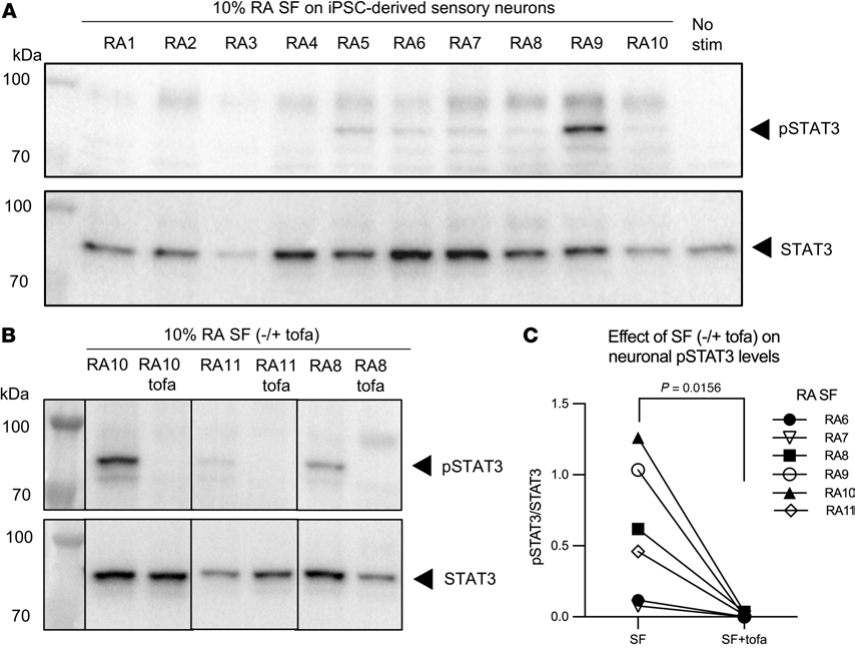
RA-derived synovial fluid can induce pSTAT3 in human sensory neuron cultures, which is blocked by tofacitinib. (**A**) IPSC-derived sensory neurons (day 55) were incubated for 1 hour with 10% RA SF (*n* = 10) or media alone (No stim). Cells were lysed and probed for pSTAT3 (upper panel) and STAT3 (lower panel) using Western blot. (**B** and **C**) IPSC-derived sensory neurons were stimulated as in **A** in the absence or presence of tofacitinib (2 μM) for 1 hour and pSTAT3/STAT3 expression analyzed. Representative Western blot (**B**) and cumulative data (**C**) showing the quantification of the ratio of pSTAT3/STAT3 signal of neurons from two batches (day 55–65) stimulated with 10% RA SF from *n* = 6 different individuals with RA in the absence or presence of tofacitinib. Data were analyzed using a paired nonparametric 1-tailed *t* test.

We tested whether neuronal STAT3 expression was functionally relevant by investigating its ability to become phosphorylated (pSTAT3, Tyr705) in the context of the inflamed RA joint environment. For this, we incubated iPSC-derived sensory neurons with a panel of paired serum and joint-derived synovial fluid (SF) donated by individuals with RA. We found that several RA SF samples were able to induce neuronal pSTAT3 albeit to varying degrees, while paired serum did not (Figure 3A and Supplemental Figure 2A). This suggests that neuronal pSTAT3 is not induced by a common feature of human biofluid but is more likely driven by specific inflammatory cytokines enriched in RA SF. Moreover, RA SF-induced pSTAT3 colocalized with BRN3A, confirming signalling specific to sensory neurons (Supplemental Figure 2B). To ascertain the role of the JAK/STAT pathway in these findings, we repeated our experiments in the absence or presence of tofacitinib, a clinically approved JAKi. We observed a complete block of neuronal pSTAT3 induction by RA SF in the presence of tofacitinib (Figure 3, B and C). Taken together, our data support the possibility that JAK/STAT3 signalling is activated in sensory neurons by SF from some individuals with RA and that JAKi can directly inhibit that activation.

We next sought to determine which cytokines may contribute to the observed neuronal pSTAT3 induction by RA SF. Given that induction was specific to SF (versus patient-matched serum), we were particularly interested in identifying JAK/STAT3-relevant cytokines that are enriched in SF. We focused our experiment on cytokines with corresponding receptors on sensory neurons, specifically type-I IFNs (IFN-α, IFN-β) and IL-6 family cytokines: LIF, which signals through the LIF receptor and gp130; IL-6 and IL-11, which signal through gp130 with their corresponding soluble or membrane-bound receptors; and Oncostatin M (OSM), which also signals through gp130 together with the LIF receptor or OSM receptor. We compared the levels of these cytokines in 12 paired serum and SF samples from patients with seropositive RA and 4 healthy control serum samples using Luminex (Table 1).

**Figure 4:**
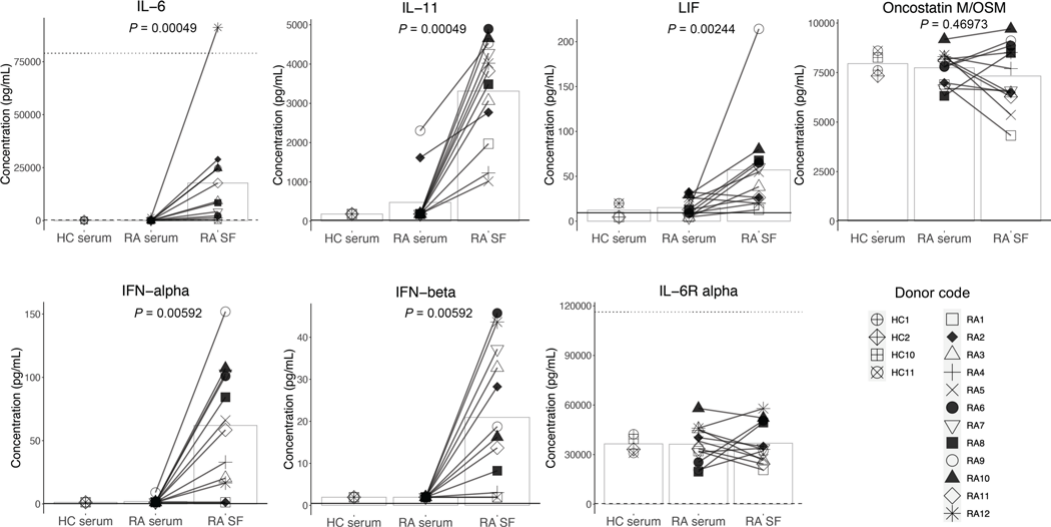
Cytokines upstream of STAT3 signaling are enriched in RA SF. Healthy control (HC) serum (*n* = 4) and paired RA serum and SF (*n* = 12) were analyzed for the presence of IL-6, IL-11, LIF, OSM, IFN-α, IFN-β, and IL-6R-α by Luminex assay. Statistical significance was assessed using paired nonparametric 2-tailed *t* tests (RA SF versus serum); *P* < 0.008 was considered significant after Bonferroni correction for the 6 cytokines. The dotted lines for IL-6 and IL-6R-α graphs indicate the highest standard value. The dashed lines indicate the lowest standard value. The legend symbols are representative of individual HC or RA samples; the bars represent the data means. Standard values, sensitivity, and all individual means (+SD) of the assay are listed in Supplemental Table 4.

We found elevated levels of IL-6, IL-11, LIF, IFN-α, and IFN-β in RA SF compared with RA serum, while OSM levels were high in both. Among these cytokines, IL-6 and IL-11 were particularly enriched in SF (Figure 4). While sensory neurons do not express membrane-bound IL-6 receptor (Figure 1, C and E), soluble IL-6 receptor (IL-6Ra) was abundantly detected in all the serum and SF samples we investigated, in agreement with a previous report (20) (Figure 4 and Supplemental Table 4). This suggests that transsignaling of IL-6 with its coreceptor gp130 could take place on neurons. We also noted that the levels of IL-6, LIF, and IFN-α in RA SF correlated well with RA SF–induced neuronal pSTAT3 levels as measured by Western blot (Supplemental Figure 3).

To identify which cell types in RA synovial tissue might express these cytokines, we examined sequencing data from the Accelerating Medicines Partnership (AMP) RA Phase-1 initiative (21). Fibroblasts, T cells, B cells, and monocytes all expressed mRNA for IL-6, LIF, and OSM, as well as IL-6 receptors and IL-11 receptors (Supplemental Figure 4). To confirm these findings at the protein level, we set up independent cultures of RA synovial fibroblasts or RA-derived mononuclear cells and tested their cell culture supernatant for the presence of these cytokines by Luminex. When RA synovial fibroblasts were stimulated with IL-1β (22), they became a rich source of IL-6, IL-11, LIF and IFN-α (Supplemental Figure 5A). Similarly, when peripheral blood mononuclear cells (PBMC) and synovial fluid mononuclear cells (SFMC) were subjected to T cell stimulatory conditions (anti-CD3 + anti-CD28 mAbs), they increased the expression of IL-6, LIF, and IFN-α (Supplemental Figure 5B). These data support the notion that activated fibroblasts and immune cells present in inflamed RA joints can be a cellular source of STAT3 signalling cytokines capable of activating sensory neurons directly.

**Figure 5:**
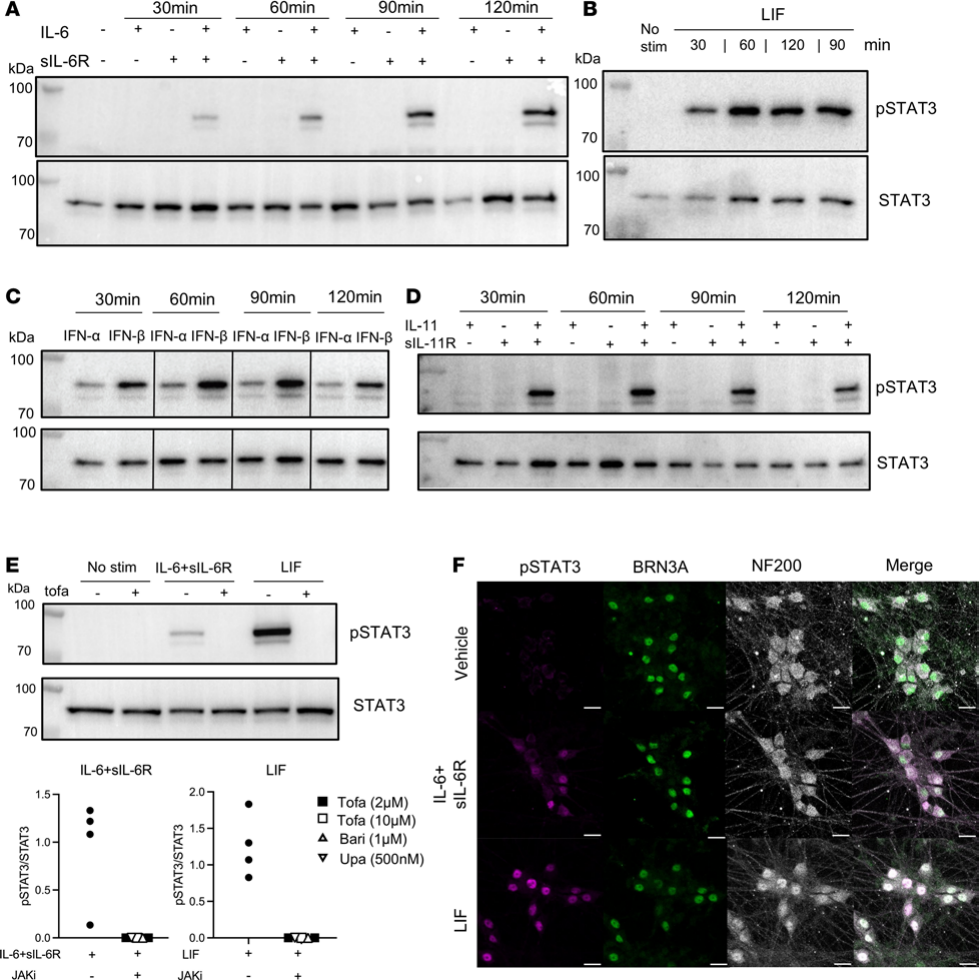
RA-relevant recombinant cytokines induced neuronal pSTAT3 in peripheral sensory neurons. (**A**–**D**) IPSC-derived sensory neurons were incubated for 30, 60, 90, and 120 minutes with human recombinant (**A**) IL-6, sIL-6R, or both (100 ng/mL), (**B**) LIF (100 ng/mL), (**C**) IFN-α (300 U/mL), IFN-β (300 U/mL), or (**D**) IL-11, sIL-11R, or both (100 ng/mL). Cell lysates were probed for pSTAT3 and STAT3 by Western blot. (**E**) Neurons from 2 batches were incubated with IL-6 + sIL-6R or LIF without or with JAKi for 1 hour. Representative blot of neurons blocked using 10 μM tofacitinib and cumulative data using tofacitinib, baricitinib or upadacitinib (*n* = 4–5). (**F**) Neurons (day 77) were stained for pSTAT3 and sensory neuron markers BRN3A and NF200 by immunocytochemistry, shown in individual or merged channels. Scale bar: 20 μm.

We next asked whether SF induction of neuronal pSTAT3 could be recapitulated with recombinant cytokines, namely IL-6 ± soluble IL-6R (sIL-6R), LIF, IL-11 ± soluble IL-11R (sIL-11R), IFN-α and IFN-β. All five mediators induced neuronal pSTAT3 at the time points investigated (Figure 5, A–D). Using IL-6 + sIL-6R and LIF as examples, we assessed whether the pSTAT3 signal could be blocked by tofacitinib. We found that tofacitinib and 2 other clinically approved JAKi, baricitinib and upadacitinib, completely blocked IL-6 + sIL-6R or LIF-induced neuronal pSTAT3 (Figure 5E and Supplemental Figure 6). To ensure that the pSTAT3 signal observed by Western blot was derived from neurons and not the occasional nonneuronal cells, we used immunocytochemistry to confirm colocalization of pSTAT3 with neuronal markers BRN3A and NF200 following stimulation with IL-6 + sIL-6R or LIF (Figure 5F). Taken together, these data indicate that cytokines enriched in the inflamed RA joint can directly induce neuronal pSTAT3, and that JAKi can block such effects.

**Figure 6:**
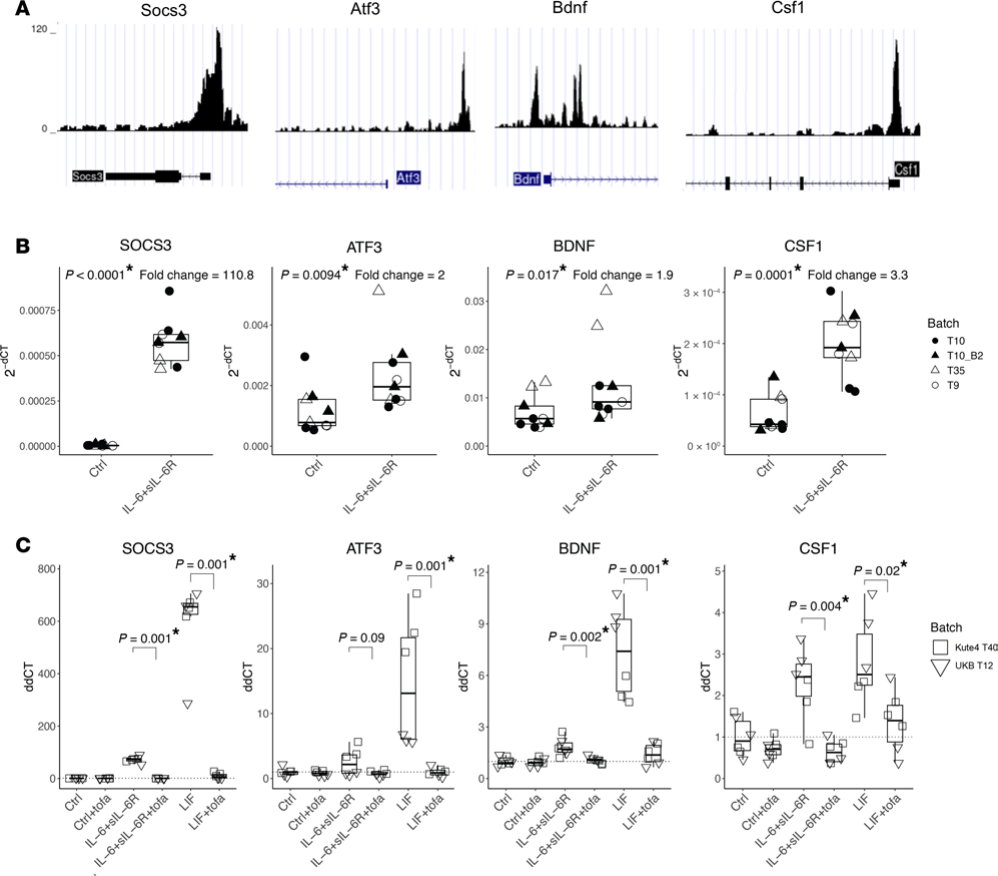
IL-6 + sIL-6R and LIF increase the expression of pain-relevant genes with pSTAT3 binding sites, which is reversed by JAKi. (**A**) ChIP sequencing dataset (23) showing pSTAT3 binding sites in *Socs3*, *Atf3*, *Bdnf*, and *Csf1* in mouse DRG neurons. (**B**) IPSC-derived sensory neurons were treated without or with 100 ng/mL IL-6 + sIL-6R for 24 hours, followed by qPCR analysis. Boxplots showing gene expression of the indicated genes. Data derived from *n* = 9 culture wells using three independent differentiations (designated by the prefix “T”) from two iPSC lines (day 67–76). (**C**) IPSC-derived sensory neurons were treated without or with IL-6 + sIL-6R or LIF (all at 100 ng/mL) for 24 hours in the absence or presence of tofacitinib (2 μM), followed by qPCR analysis. Boxplots showing expression of the indicated genes. Data are derived from *n* = 6 neuronal wells derived from two independent iPSC lines (day 57–61). Statistical significance was determined by 1-tailed nonparametric *t* tests to compare between with or without IL-6 + sIL-6R (**B**) or with or without tofacitinib in the presence of IL-6 + sIL-6R or LIF (**C**). Neurons aged day 57–76 were used. *P* < 0.013 was deemed significant after Bonferroni correction and marked by an asterisk.

We then explored the downstream intracellular consequences of STAT3 activation in sensory neurons. We reanalyzed a publicly available chromatin-immunoprecipitation–seq (ChIP-seq) dataset generated in mouse DRG neurons to identify possible pSTAT3 binding sites of four genes of interest (23) (Figure 6A). As expected, sensory neurons showed pSTAT3 binding in the promoter region of SOCS3, the negative regulator of STAT3 activation (24). Of interest to sensory neuron function, there also appeared to be pSTAT3 binding sites in several well-known pain-relevant genes: ATF3, an injury and regeneration marker (25, 26); BDNF, a growth factor that mediates central sensitization (27, 28); and CSF1, a cytokine that promotes microglia and macrophage activation in DRG and spinal cord, likely exacerbating pain (29, 30). To confirm involvement of these genes in our human iPSC-derived sensory neuron system, we probed for changes in their gene expression after 24 hours of stimulation with IL-6 + sIL-6R (Figure 6, B and C). An increase in expression was observed for all four genes, as would be expected upon pSTAT3 binding. A similar and even more robust pattern was seen with LIF stimulation, and, importantly, transcriptional regulation was entirely reversed by the addition of a JAK inhibitor (Figure 6C). Together, these data reveal that JAKi can directly dampen upregulation of pain-relevant genes in sensory neurons, pointing towards a potential mechanistic explanation for why JAKi are particularly effective at reducing pain in RA.

**Figure 7:**
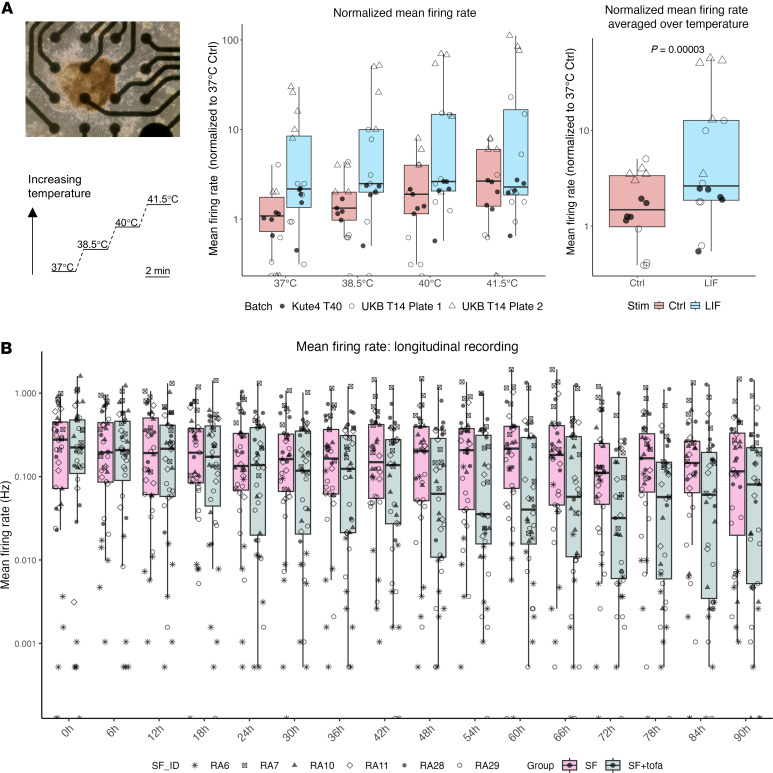
Modulation of neuronal firing by LIF and JAK inhibitors. (**A**) Neurons plated on an MEA plate (scale bar: 20 μm) treated without or with 100 ng/mL LIF for 92 hours and subjected to increased temperature. Boxplots of Ctrl (red) and LIF (blue) showing normalized mean firing rates at each temperature (middle) or averaged across temperature (right). The *P*-value derives from a nonparametric *t*-test to assess the effect of LIF. Legend symbols represent wells from 3 plates and 2 iPSC lines (*n* = 14–16, neuronal age: day 64–75). (**B**) Neurons plated on an MEA plate treated with 10% RA SF without or with 2 μM tofacitinib (tofa) for 92 hours (92 h). Boxplots of RA SF without tofa (magenta) and with tofa (grey) showing mean firing rates of neurons every 6 hours (h) from the addition of RA SF (baseline, 0h) to 90 hours (90h). A repeated-measures ANOVA was used to assess the effect of time, group (SF vs SF+tofa) and any interaction. Legend symbols represent wells from 2 plates from the Kute4 iPSC line (T52, T55) with the respective RA SF stimulation (Plate 1 - RA6, 28, 29; Plate 2 – RA7, 10, 11. *N* = 5–7 per SF ± tofa, neuronal age D54-59).

Since LIF is a lesser-known pain mediator compared with IL-6, we assessed whether LIF could affect neuronal excitability. Acute application did not increase neuronal firing rate initially; however, an increase was observed over time, at around 92 hours (Supplemental Figure 7, A and B). At this timepoint, we subjected the neurons to a temperature gradient ramping up from 37°C to 41.5°C (Figure 7A and Supplemental Figure 7, C and D). We observed a nominal increase in sensory neuron firing in response to increasing temperature, although this was not statistically significant. However, we observed a significant main effect of LIF (repeated measures ANOVA, *P* = 0.045) in modulating neuronal firing rate across the temperature range. This was even more apparent when mean firing rates were averaged over the temperature steps (*P* = 0.00003).

As a final step, we subjected neurons to RA SF in the absence or presence of the JAK inhibitor tofacitinib. We observed a significant effect of the interaction between group (SF ± tofa) and timepoints (Figure 7B, repeated measures ANOVA, *P* = 0.029). We observed considerable variability between neurons treated with different RA SF, likely reflecting patient sample heterogeneity; nonetheless, tofacitinib appeared to reduce the firing for most of the individual RA SF tested (Supplemental Figure 8). We also subjected neurons to a temperature gradient from 32°C to 45°C at both baseline and 92 hours. In both instances, neurons increased their firing rate with the large temperature steps (Supplemental Figure 9, Repeated measure ANOVA, *P* < 0.0001). There was a trend for reduced firing in the tofacitinib group at 92 hours, which was not observed at baseline (Supplemental Figure 9).

## Discussion

JAKi are frequently used in the treatment for RA due to their effectiveness, affordability, and convenience of administration (5). In light of evidence that JAKi may be more effective than anti-TNF in reducing pain in RA (7, 8), we tested the hypothesis that JAKi have a direct inhibitory effect on JAK/STAT signalling in human iPSC-derived sensory neurons.

We showed that SF taken from the inflamed joints of individuals with RA can induce neuronal pSTAT3, which can be blocked by JAKi. Our data suggest that this effect is likely due to the presence of IL-6 family cytokines (including IL-6, IL-11, and LIF) and/or type-1 IFNs in the SF. We further showed that the corresponding recombinant cytokines recapitulated neuronal pSTAT3 induction, which was blocked by JAKi. Some of these cytokines (IL-6, IFN-α) are already known pain mediators (31–34). We provided initial evidence that LIF is another cytokine that can induce neuronal sensitization. The activation of pSTAT3 induced by IL-6 + sIL-6R or LIF increased the expression of pain-related genes in iPSC-derived sensory neurons, which was blocked by JAKi. Finally, JAKi reduced neuronal firing when iPSC-derived sensory neurons were stimulated with RA SF. We hence propose that JAKi may have dual action, dampening both inflammation and neuronal activation in RA joints.

Our study adds to the pool of knowledge on the presence and abundance of inflammatory cytokines in RA SF and serum. Consistent with previous reports, we found a higher concentration of IL-6, IL-11, LIF, and IFN-α in RA SF compared with paired serum (35–39). Precise levels of SF cytokines varied between individuals, with samples with higher cytokine concentrations better able to induce neuronal pSTAT3 (Supplemental Figure 3). To our knowledge, this is the first time that levels of IFN-β and OSM were measured and compared in HC serum, RA serum, and paired SF. The level of OSM was high and similar across all samples, while IFN-β was elevated in RA SF compared with RA serum. Future studies now need to correlate the concentration of these STAT3 cytokines with joint pain intensity. From our current results, we would predict that high STAT3 cytokine levels result in more pain for individuals living with RA.

Our study presents evidence of a direct effect of some of these cytokines (IL-6, LIF, IL-11 and type-1 IFNs) on sensory neurons. STAT3 signalling machinery is expressed across all subtypes of human nociceptors (Figure 1A), suggesting that peptidergic and nonpeptidergic nociceptors, as well as nociceptive A-δ fibres, have the potential to be equally impacted by JAK/STAT activation. Our findings are corroborated by two recent publications in rodent models that showed that IL-6 + sIL-6R can elevate joint sensory neuron firing in vivo and in vitro, as well as elevate *Socs3* and *Csf1* in a mouse neuroblastoma cell line (40, 41). In keeping with our results, both papers also indicate that the observed effects can be reversed by administration of JAKi (40, 41).

Of the JAK/STAT cytokines we studied, IL-6 and type-1 IFN are already known to be able to elevate neuronal excitability in vivo and in primary mouse DRG neurons (31–33). Here, we provide initial evidence that the same is true for LIF, which appeared to be able to modulate the firing rate of iPSC-derived sensory neurons in response to temperature and was enriched in RA SF. One prior study reported that, compared with SF taken from individuals in a non-arthritic control group, SF from patients with osteoarthritis increased excitability in mouse DRG neurons (42). Notably, osteoarthritis SF is also reportedly enriched in STAT3-activating cytokines such as IL-6 and IL-11 (43). Here, we focused on the inhibitory effect of JAKi. We found that tofacitinib reduced neuronal firing in the presence of RA SF, supporting the notion that cytokines from RA SF can directly activate neurons.

Our findings are limited by the fact that the RA SF samples we used were all collected from female patients with active, seropositive RA. While this represents a very prevalent population among individuals with RA, it does not reflect the full heterogeneity of those affected by the disease. Whether a similar pattern is seen in seronegative RA, in male patients, or in those with low disease activity, remains to be tested. Moreover, for future studies, it would be informative to capture information on pain scores and correlate them with JAK/STAT3 signalling in sensory neurons.

Indeed, with pain being one of the top concerns for individuals living with RA, it is important to understand exactly which JAK/STAT cytokines within RA SF are the most potent modulators of sensory neuron activity. Here, we identified multiple candidates that could be exploited in drug development, e.g., one could consider blocking cytokine signalling upstream of STAT3 using bispecific antibodies targeting specific IL-6 family cytokines such as IL-6, LIF, and IL-11 (44, 45).

Future work in this area will not only be relevant to RA, but also to other immune-mediated inflammatory diseases where the JAK/STAT pathway is known to be involved in pathogenesis, such as psoriatic arthritis and inflammatory bowel disease (46). Our work raises the possibility that in all of these instances, inhibiting JAK/STAT3 signalling might limit sensory neuron hyperexcitability not only indirectly by dampening inflammation, but also directly by interfering with intracellular pathways in neurons. We hope that this mechanistic insight can accelerate the development of novel painkillers to help improve the quality of life of the millions of individuals currently living with pain induced by RA and other, pathologically similar conditions.

## Methods

### Sex as a biological variable.

Our study utilized female (Kute4) and male (UKB) iPSC lines to derive sensory neurons, and findings using this model may be applicable to both sexes. The serum and synovial fluid used for Luminex experiments were derived from female donors only, as RA predominantly affects women. Findings on the characterization of mediator profiles may be applicable to female patients only.

All reagents and antibodies used, including their concentrations and catalogue numbers, are listed in Supplemental Table 1. Fibroblasts, PBMC/SFMC culture, sensitivity analyses, and bioinformatic analyses are described in the Supplemental Methods.

### IPSC culture, sensory neuron differentiation, and maintenance.

Two iPSC lines, Kute4 (HPSI0714i-kute_4, female) and UKB (UKBi013-A-GCamP6f, male (17) were used in this study. IPSC were maintained in Stemflex media on vitronectin-coated wells and passaged using versene when reaching approximately 70% confluency. IPSC were differentiated into sensory neurons once reaching approximately 60% confluency following the Chambers protocol and replated at 75,000 cells per coverslip in a 24-well plate at day 11 (19, 47). Neurons were cultured in N2 media supplemented with NGF, NT-3, GDNF, and BDNF at 25 ng/mL (day 11–39) or 10 ng/mL (day 39 onwards). 1–3 μM AraC was supplemented to inhibit the growth of nonneuronal cells based on morphological examination around day 14 to day 30. Geltrex (1 in 250 dilution) was supplemented once every week to stabilize the culture and prevent peeling.

### Human samples.

For healthy control samples, following written informed consent, a maximum of 100 mL peripheral blood was drawn from healthy donors into Vacutainer tubes (BD Biosciences) or, in some instances, leucocyte cones from healthy volunteer blood donations were purchased from the NHS Blood and Transplant service. Peripheral blood samples and synovial fluid samples from patients with RA were collected with informed consent from patients attending Guy’s Hospital Rheumatology Department. RA synovial fibroblasts were derived from joint replacement surgery or synovial tissue biopsies and cryopreserved in liquid nitrogen. Details of PBMC, SFMC, and synovial fibroblast isolation and cultures can be found in the Supplemental Methods. Cell-free serum was obtained by centrifuging a serum tube at 3,000*g* for 10 minutes and collecting the supernatant. Cell-free SF was obtained by centrifuging SF at 5,900*g* for 3.5 minutes and extracting the supernatant. Cell-free serum and SF aliquots were stored at –80°C.

### Neuron stimulation.

Neurons past differentiation day 50 were used in this study. For SF stimulation, SF was centrifuged at 16,000*g* for 4 minutes and diluted to 10% in N2 media. For cytokine stimulation, neurons were incubated in the cytokines for the time and concentration specified in the figure legends. In experiments using JAKi, neurons were preincubated with N2 media containing tofacitinib (tofa), baricitinib (bari), or upadacitinib (upa) for 1 hour before cytokine stimulation. IL-6 and sIL-6R, and IL-11 and sIL-11R were premixed for 1 hour prior to use in experiments. The cytokine stimulation media also contained the same concentration of JAKi to ensure continuous blockade.

### Immunocytochemistry.

Neuron-containing coverslips were fixed in 4% paraformaldehyde (PFA) at room temperature for 10 minutes. Purity staining (BRN3A and PGP9.5) was performed on neurons blocked with 10% donkey serum in 0.1% PBS-Triton for 1 hour, then incubated with primary antibodies overnight at room temperature (BRN3A, PGP9.5). After washes, secondary antibodies were applied for 2 hours at room temperature. To stain for pSTAT3, BRN3A, and NF200, following PFA fixation, cells were further permeabilized in 100% methanol at –20°C for 10 minutes, followed by blocking in 5% donkey serum in 0.3% PBS-Triton for 1 hour and incubation with primary antibodies overnight (pSTAT3, BRN3A, and NF200) at 4°C. Secondary antibodies were applied for 2 hours, followed by a final incubation in horse-anti-rabbit streptavidin for 30 minutes. PBS washes were performed between each step. Coverslips were mounted on DAPI containing mounting media. The proportion of BRN3A^+^ neurons was quantified using a bespoke script developed in Fiji (version 2.14) (17, 48).

### Western blot.

Neurons were washed with ice-cold PBS before harvesting in RIPA buffer with phosphatase and protease inhibitors. In cases of RA serum/SF stimulation, neurons were washed twice to minimize residual protein carry over. BCA assays were performed to measure protein concentration to normalize protein for loading.

Samples were mixed with 4 × Laemmli buffer and boiled at 95°C for 5 minutes. Samples and a protein ladder were loaded onto Novex Tris-Glycine Mini Protein Gels and run at 100 V for 1.5 hours. Protein was transferred from the gel to methanol-activated 0.45 μm PVDF membranes using the Trans-Blot Turbo Transfer kit. Membrane blocking was performed at room temperature for 1 hour in 5% skimmed milk, dissolved in TBS with 0.1% Tween 20 detergent (TBST), and then probed with primary anti-pSTAT3 antibody overnight at 4°C. After three 5-minute washes in TBST, membranes were incubated with secondary Ab (diluted in TBST) for 1 hour. After washing, blots were imaged using SuperSignal Chemiluminescent Substrates. Data were acquired by Chemidoc XRS+ and Image Lab software (version 6.1). Blots were stripped in Millipore stripping buffer for 15 minutes, then blocked and incubated with primary anti-STAT3 antibody, following the same steps as above. Densitometric analyses were conducted in Fiji (version 2.14) to calculate pSTAT3/STAT3 ratios.

### RNA extraction, cDNA conversion, and qPCR.

Neurons were lysed in RLT buffer with β-mercaptoethanol and RNA extracted using a Qiagen RNeasy Kit. RNA concentration was measured using a Qubit High Sensitivity kit. The same amount of RNA was converted to cDNA using SuperScript III and 2 ng/mL cDNA was used for qPCR using the Roche LightCycler FastStart DNA Master SYBR Green mix. Primers used in the study are listed in Supplemental Table 2. ΔCycling Thresholds (CT) were obtained by normalizing CT values to the housekeeping genes. The data are presented as either 2^–ΔCT^ or fold change (ΔΔCT), which was calculated by dividing 2^–ΔCT^ in each condition by the average 2^–ΔCT^ of control.

### Flow cytometry.

IPSC were detached from the well using TrypLE, washed with PBS, then stained with fixable Viability Dye for 20 minutes at 4°C. Cells were washed with FACS buffer (1% BSA, 0.1% sodium azide in PBS) at 5,900*g* for 3.5 minutes and blocked in 10% human Ab serum in FACS buffer for 10 minutes at 4°C. TRA-1-60 mAb was used to stain iPSC for 30 minutes at 4°C. After a final wash, iPSC were fixed with 2% PFA for 15 minutes at 4°C before being washed a final time and resuspended in FACS buffer. Unstained cells and live/dead controls were prepared to optimize fluorochrome acquisition. Unstained cells were taken through the same workflow as stained cells, without the inclusion of antibodies. For live/dead controls, half of the cells were heat inactivated at 65°C for 5 minutes followed by a cold shock on ice for 5 minutes, then combined with another half of live iPSC and stained with fixable Viability Dye. Samples were acquired using a FACS Canto-II. Data were analyzed using FlowJo software (version 10.8).

### Luminex.

A Luminex Discovery Assay (R&D) was used to determine the concentration of IL-6, IL-6R-α, Oncostatin M (OSM), IFN-α, IFN-β, IL-11, and LIF. Samples were diluted 1:2 (cell culture media, serum, and SF) and 1:100 (serum and SF). The plate signal was acquired using FLEXMAP 3D and xPonent software. Experiments and analyses were carried out following manufacturer’s instructions. A five-parameter logistic (5-PL) curve fit was performed to establish standard curves. When the value could not be determined by the standard curve, the highest or lowest value obtained for the analyte was assigned to the sample.

### Multielectrode array recording and analyses.

Neurons were replated at day 11 on Axion Cytoview 24-well plates or Cytoview 48-well plates coated with 0.1% PEI (diluted in 1x borate buffer) and 8 μL 1/50 Geltrex (diluted in Knockout DMEM), at 50,000 cells per well. The spontaneous activity of mature neurons (older than day 50) was recorded using the Axion Maestro Edge machine (Axion Biosystems) and Axis Navigator software (version 3.9.1). Before the experiment, neurons were acclimatised with starvation media (Neurobasal+glutamax) for at least 4 hours, then recorded at baseline. Cytokines or vehicle (0.1% BSA) were added 1 hour later and neurons were recorded every 6 hours for 2 minutes until 90 hours. For the LIF experiments (Figure 7A), at 92 hours, a temperature ramp was applied from 37°C to 41.5°C, with 1.5°C increments and held at each step for 2 minutes. A well was considered active if there were more than 4 spikes detected in the four temperature steps analyzed. For the RA SF (± tofacitinib) experiments (Figure 7B), neurons underwent a baseline temperature ramp from 32°C to 45°C, with 2-minute recordings at 32°C, 37°C, and 45°C, along with continuous recording during temperature increases. Neurons were evenly assigned to either the SF or SF + tofacitinib groups based on their firing rate at 37°C during the baseline temperature ramp. Neurons were rested for 1 hour before adding 2 μM tofacitinib and subsequently incubated for another hour. RA SF (± tofacitinib) was then applied, and neurons were recorded from 0 hours (10–30 minutes postaddition) to 90 hours, every 6 hours. The RA SF used in multielectrode array *(*MEA) were tested for their ability to induce neuronal pSTAT3 in separate Western blot experiments. A final temperature ramp was conducted at 92 hours. Continuous data were acquired simultaneously at 12.5 kHz per electrode and filtered using a 1-pole Butterworth band-pass filter (200–3,000 Hz) every 6 hours. Individual spikes were counted if they crossed a 5.5 SD threshold.

### Statistics.

Statistical tests and sensitivity analyses were performed using Microsoft Excel, R (version 4.1.2) or G*Power (version 3.1.9). The statistical tests used are specified in the figure legends. To assess the effect of JAKi on RA SF induced pSTAT3, we used the 1-tailed paired nonparametric *t* test, with *P* < 0.05 considered significant. To compare cytokine levels in RA serum and SF, we used 2-tailed paired nonparametric *t* tests, where *P* < 0.008 was considered significant following multiple comparison correction. To assess the effect of pSTAT3 activation or JAKi on neuronal transcription, we used 1-tailed nonparametric *t* tests, where *P* < 0.0125 was considered significant following multiple comparison correction. We used repeated measures ANOVA to assess the effect of LIF or JAKi on mean firing rates across temperature or time, with *P* < 0.05 as the significance threshold in all cases. Data are presented in scatter plots, bar graph (indicating the mean) or standard boxplots. Sensitivity analyses can be found in the Supplemental Methods. Plots were generated in GraphPad Prism 10 or using ggplot2 in R studio (49). Details of all the statistical tests performed in this study (including n numbers, units of analyses and observed effects) are summarized in Supplemental Table 3.

### Study approval.

Ethics approval for the study and sample collection was obtained from the Bromley Research Ethics Committee (06/Q0705/20) and Harrow Research Ethics Committee (17/LO/1940).

### Data availability.

All data underlying graphs in the main and supplemental figures are provided in the Supporting Data Values file. Sequencing analyses were conducted on public repositories specified in the main text and figure legend. Accession codes are available in the original publications (13, 14, 17).

## Author contributions

YL conducted the experiments and drafted the manuscript. EHG and RR conducted immune cell and fibroblast experiments for culture supernatant generation. LJK, IZ, AL, and LF helped with growing iPSC-derived sensory neurons. RJM supported Western blot experiments. SR processed and catalogued the serum, blood, and synovial fluid samples. PR and OB generated the UKB-GCamP6f iPSC line. SJ provided intellectual support. FD and LST conceptualised and co-supervised the project, helped draft the manuscript and provided intellectual support. All authors edited and/or reviewed the final draft of the manuscript.

## Supplementary Material

Supplemental data

Unedited blot and gel images

Supporting data values

## Figures and Tables

**Table 1:**
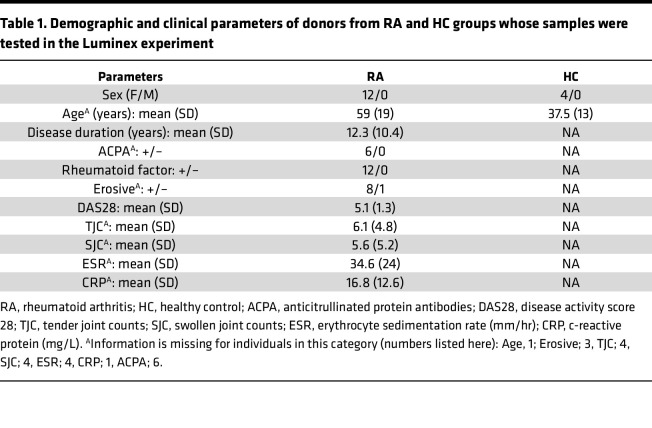
Demographic and clinical parameters of donors from RA and HC groups whose samples were tested in the Luminex experiment

## Version 1 05/15/2025

In-Press Preview

## Version 2 06/23/2025

Electronic publication
