# Supplementary material for: Rheumatoid arthritis synovial fluid induces JAK-dependent intracellular activation of human sensory neurons
Source: JCI Insight. 2025 May 15;10(12):e186646. doi: 10.1172/jci.insight.186646 (PMC12220938; doi:10.1172/jci.insight.186646)
Supplement: Supplemental data [file jciinsight-10-186646-s127.pdf]

Supplementary figures

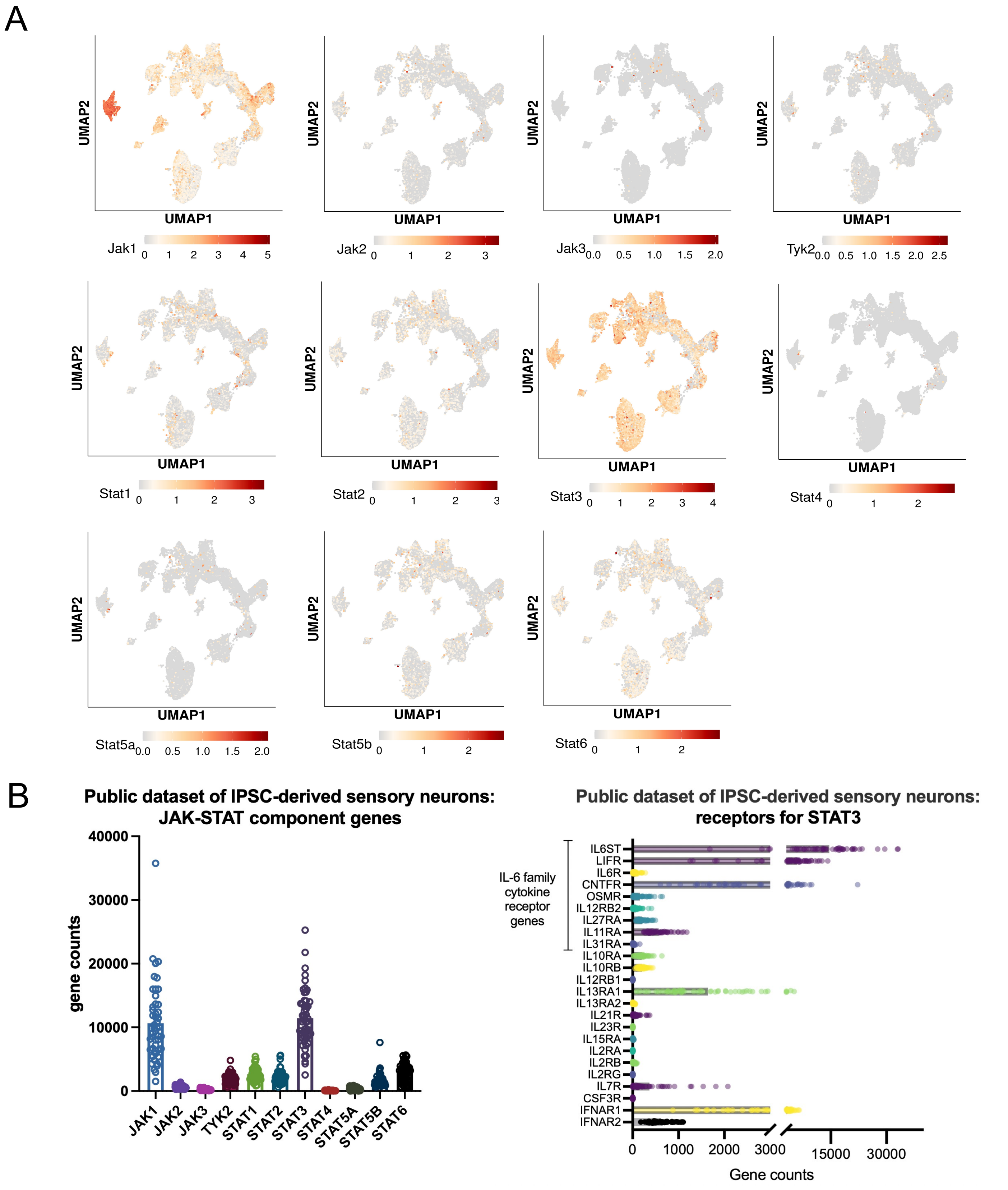

Supplementary Figure 1. JAK1 and STAT3 are the two most highly expressed components in human and iPSC sensory neurons. A. Analyses of a compilation atlas also showed that JAK1 and STAT3 are abundantly expressed by human sensory neurons compared to other JAKs and STATs (15). **B.** A public RNA-seq dataset of iPSC-derived sensory neurons (18) shows very similar expression patterns of JAK/STAT signalling components compared to our in-house iPSC derived sensory neurons (17).

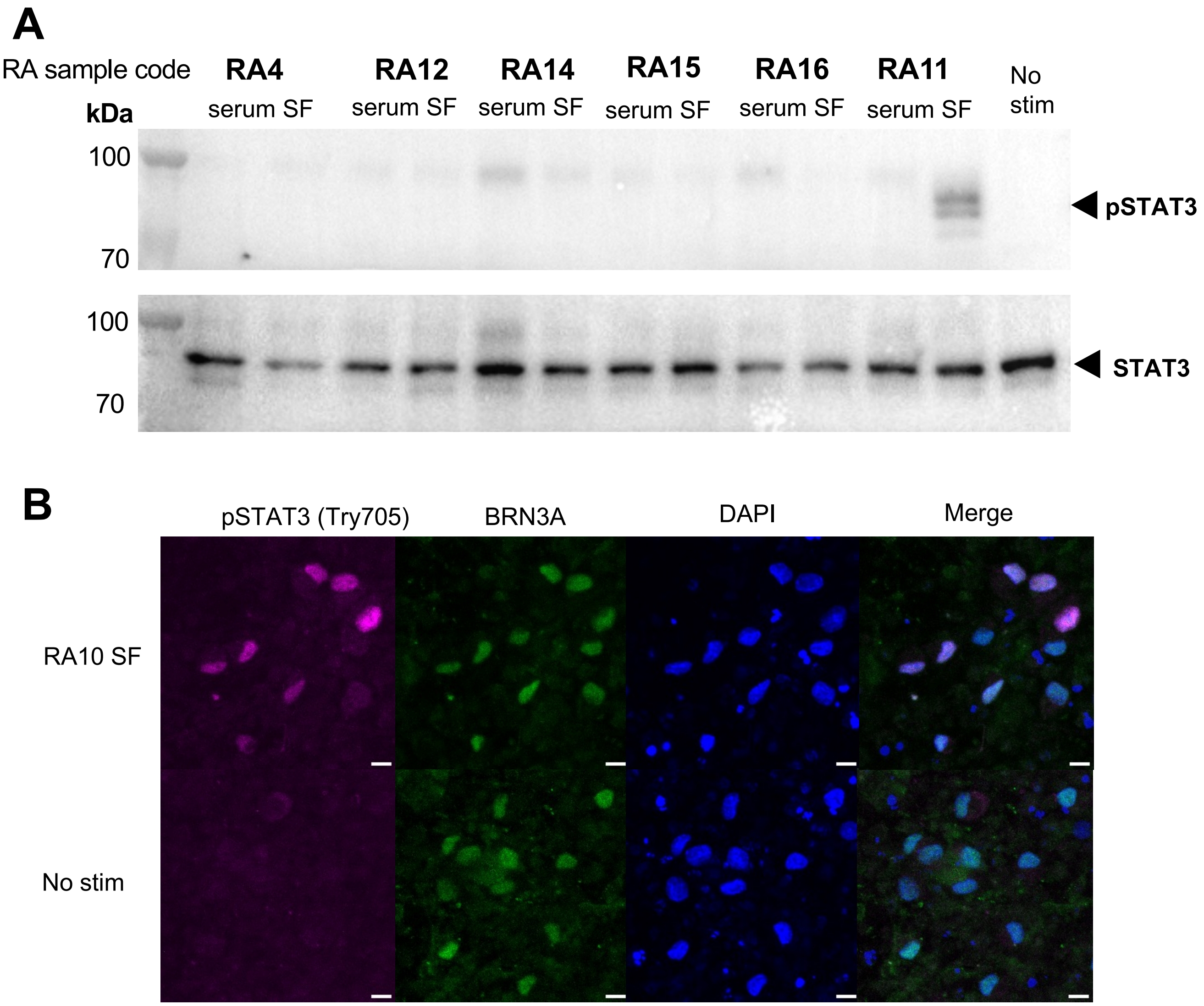

Supplementary Figure 2. RA SF can induce neuronal pSTAT3.

A. RA SF but not paired serum induced pSTAT3 in iPSC-derived sensory neuron cultures. Both SF and serum were used at 10% and applied to sensory neurons for 1 hour before neurons were lysed for Western blot.

B. Immunocytochemistry of human iPSC-derived sensory neurons stimulated with 10% SF or media only for 1 hour. There is a strong pSTAT3 signal in the nuclei of more than half of the sensory neurons following 1 hour stimulation. Scale bar: 10μm.

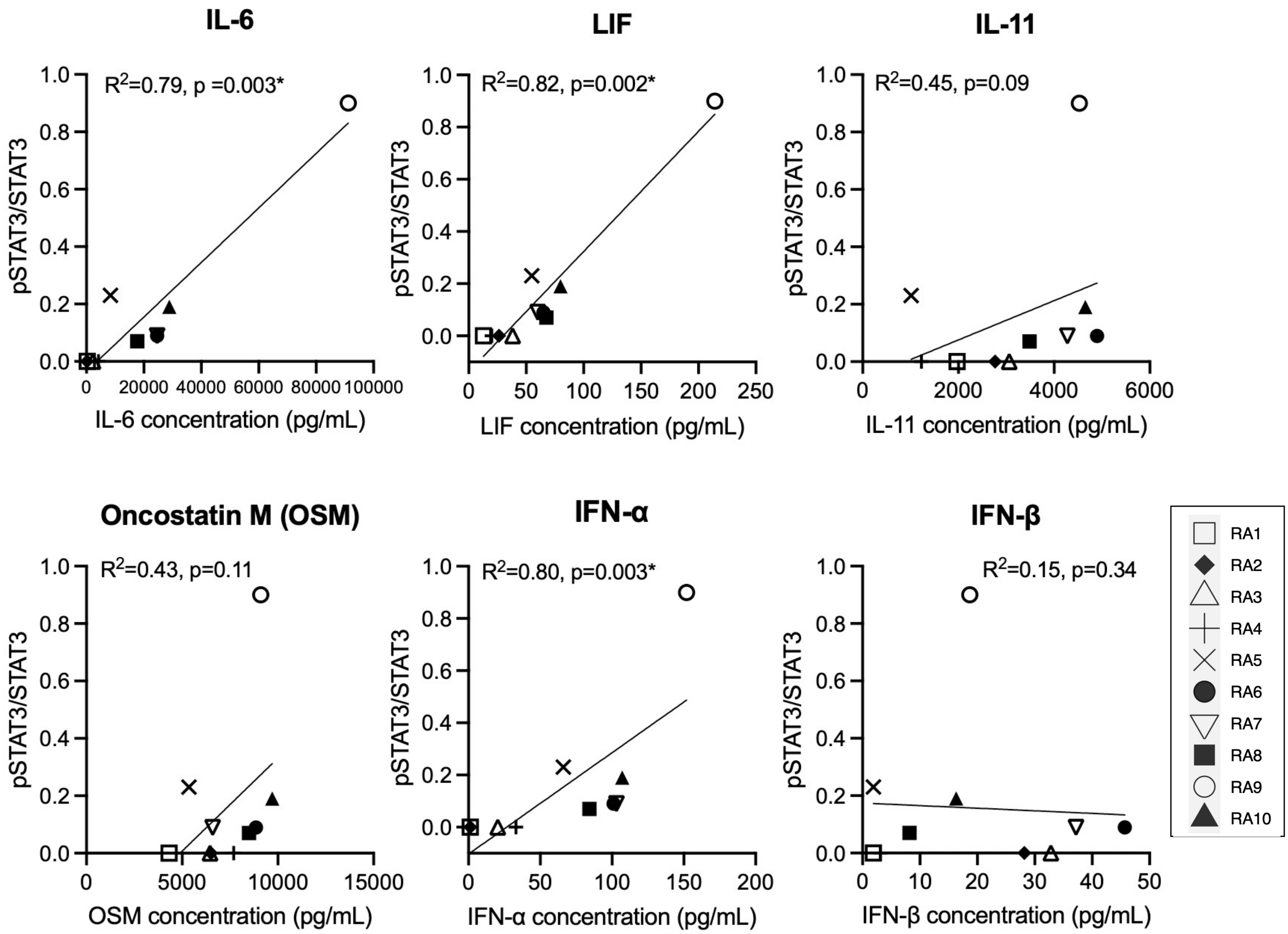

Supplementary Figure 3. Neuronal pSTAT3 levels induced by RA SF correlated with the cytokine levels in RA SF. Spearman's correlations of the level of STAT3 cytokines in RA SF (shown in Figure 4) and the neuronal pSTAT3/STAT3 they induced (n=10). Significance after multiple comparison correction indicated by * (cut-off: p=0.008 for 6 cytokines).

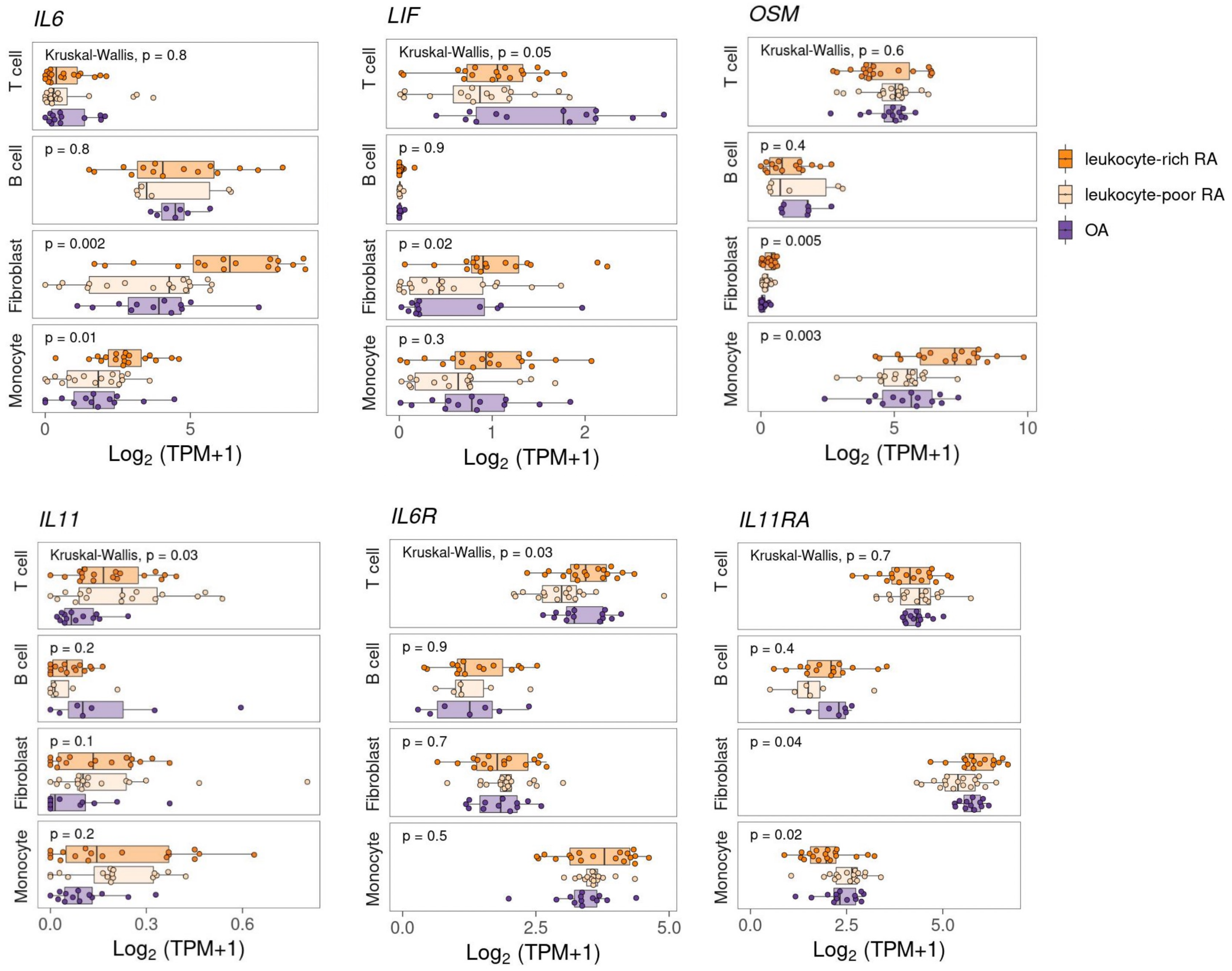

Supplementary Figure 4. Putative cellular source of STAT3 cytokines. AMP-1 bulk RNA-seq analyses reveal immune cells and fibroblasts produce IL-6, LIF, IL-11, OSM, IL-6R and IL-11RA at mRNA level (21) (https://immunogenomics.io/ampra/).

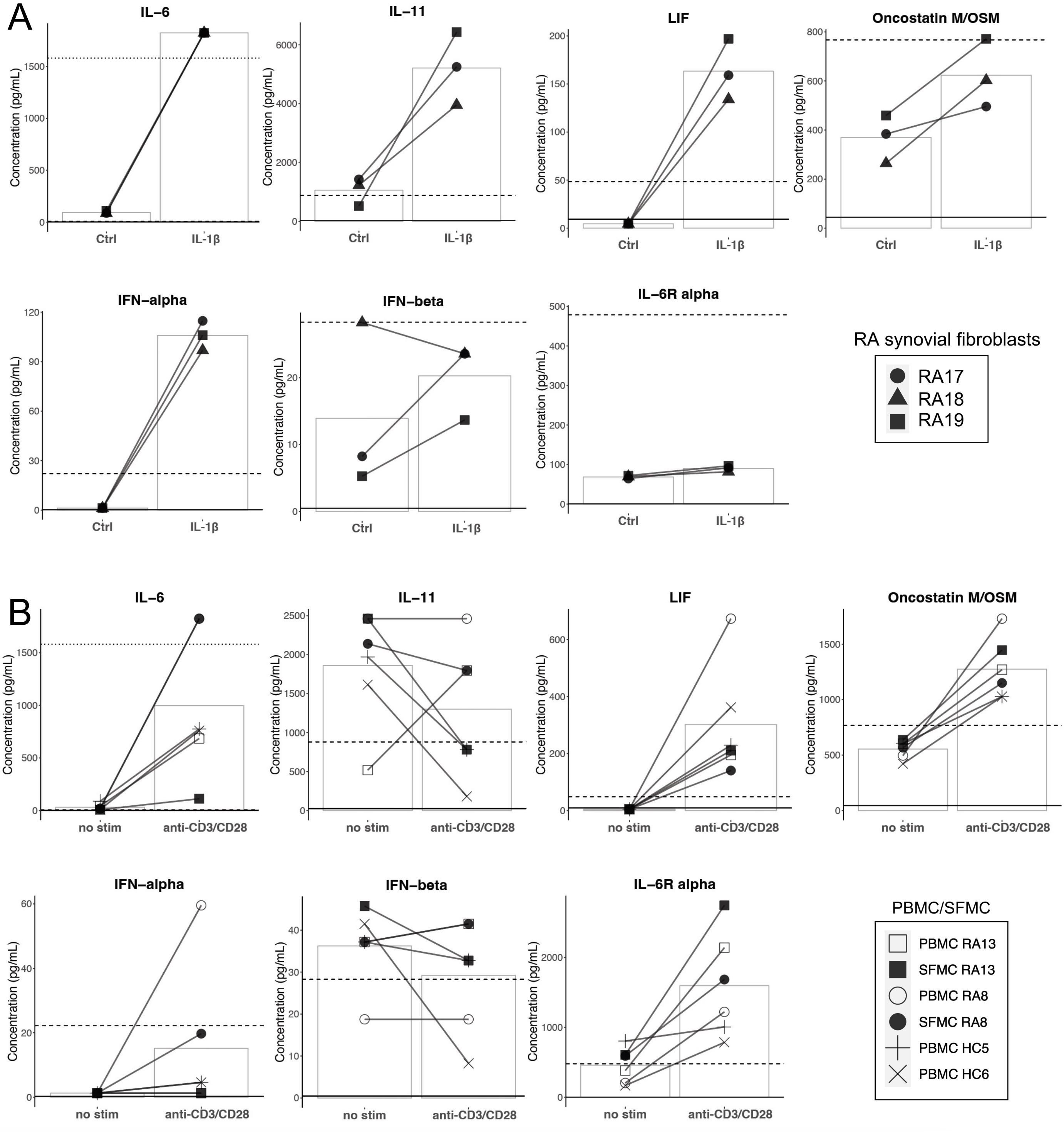

Supplementary Figure 5. RA synovial fibroblasts and RA PBMC and SFMC are putative sources of IL-6, IL-11, LIF and IFN-alpha. A. RA synovial fibroblasts showed increased expression of IL-6, IL-11, OSM and IFN-alpha following 10ng/mL IL-1β stimulation for 24 hours. N = 3 samples from three individuals with RA. Passage 5-6 fibroblasts were used in the experiments. **B.** PBMC and SFMC showed increased expression of IL-6, LIF, OSM following T cell stimulation (1.25 µg/mL anti-CD3 & 1 µg/mL anti-CD28) for 3 days. N = 2 paired samples from two individuals with RA, n = 2 samples from HC donors.

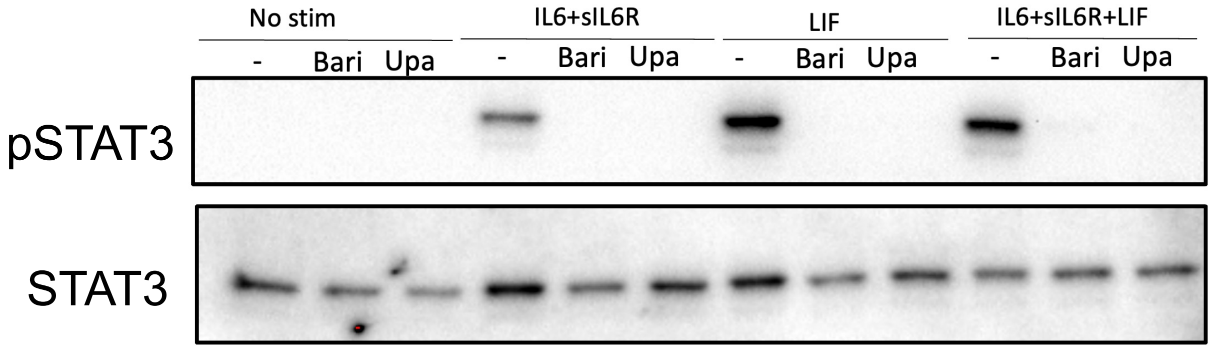

Supplementary Figure 6. Neuronal pSTAT3 can be blocked by other clinically approved JAKi. Pre-incubation of neurons with baricitinib (1uM) and upadacitinib (0.5uM) for 1 hour completely blocked neuronal pSTAT3 induced by IL-6+sIL-6R and/or LIF (IL-6, sIL-6R, LIF: 100 ng/mL). Neurons were harvested 1 hour after cytokine stimulation.

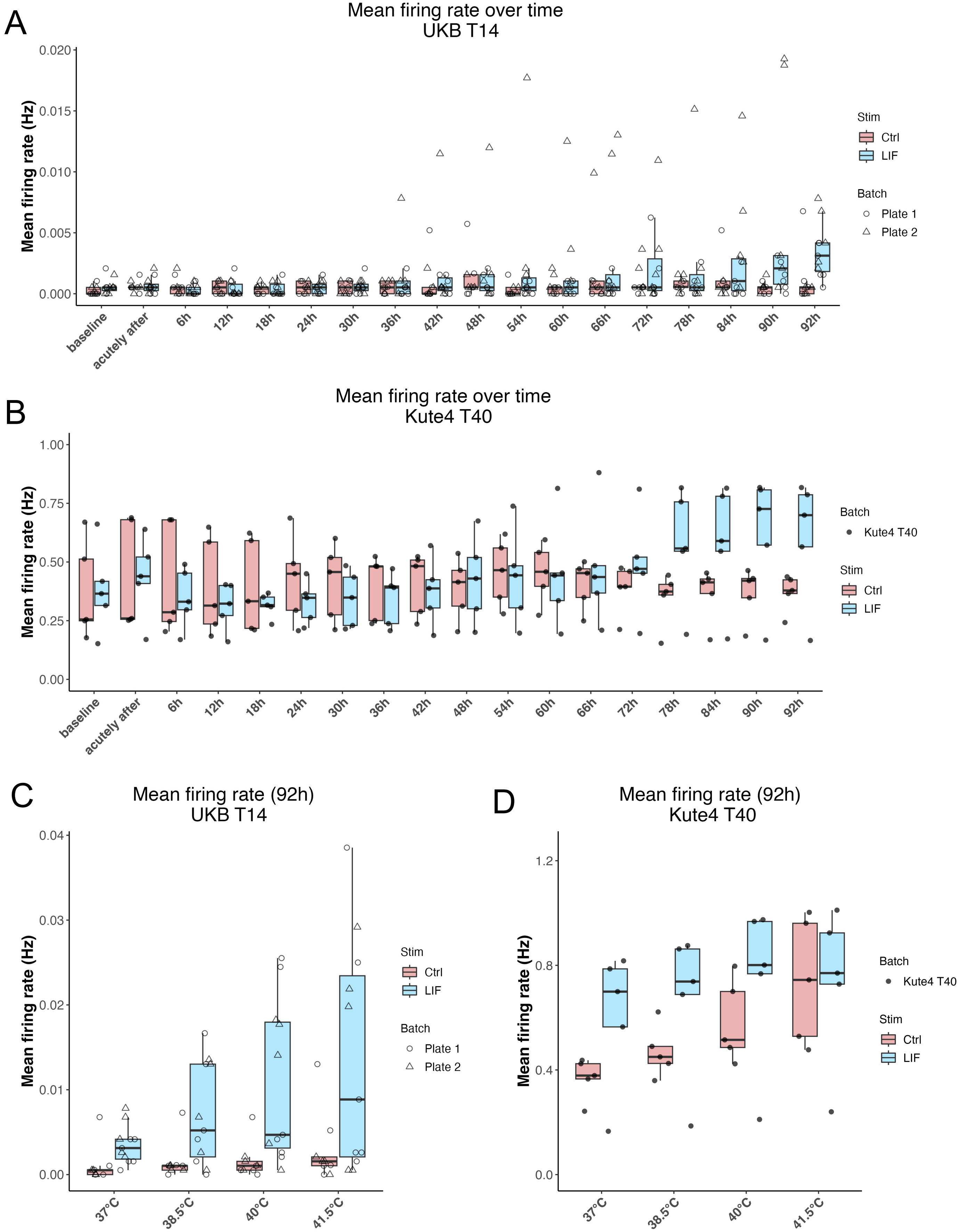

Supplementary Figure 7. Multi-electrode array analyses of iPSC-derived sensory neurons over time and across temperature steps. A-B: Longitudinal mean firing rate of UKB T14 neurons (**A**) and Kute4 T40 neurons (**B**) recorded over 92 hours. **C-D:** Boxplots showing mean firing rate of UKB T14 neurons (**C**) and Kute4 T40 neurons (**D**) at temperature steps from 37°C to 41.5°C. Neuronal age was D60, D67 (UKB T14 Plate 1&2) and D71 (Kute4 T40) at baseline. UKB T14 Plate 1&2: Ctrl (n=9) vs LIF (n=11). Kute4 T40 Ctrl (n=5) vs LIF (n=5).

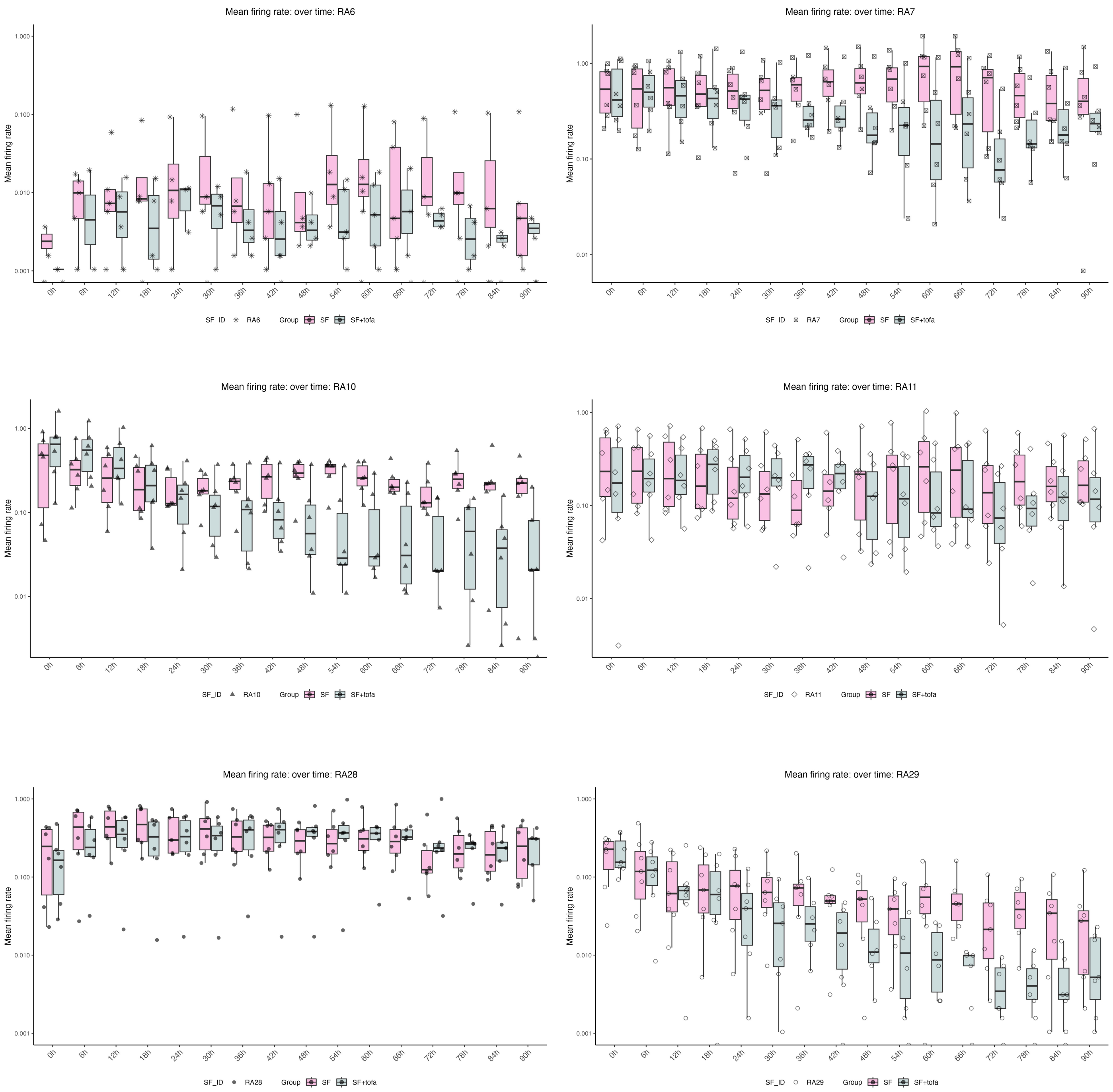

Supplementary Figure 8. JAK Inhibitors reduce firing in RA SF-Treated Neurons. Multi-electrode array analyses of iPSC-derived sensory neurons incubated with RA SF without or with JAK inhibitor tofactinib (tofa, 2μM) over time. Individual plots represent neurons incubated with distinct RA SF (10%). Boxplots of RA SF without tofa (magenta) and with tofa (grey) showing mean firing rates of neurons every six hours (h) from the addition of RA SF (baseline, 0h) to 90 hours (90h). Legend symbols represent wells from two plates from the Kute4 iPSC line (T52, T55) with the respective RA SF stimulation (Plate 1 - RA6, 28, 29; Plate 2 – RA7, 10, 11. N=5-7 per SF +/- tofa, neuronal age D54-59).

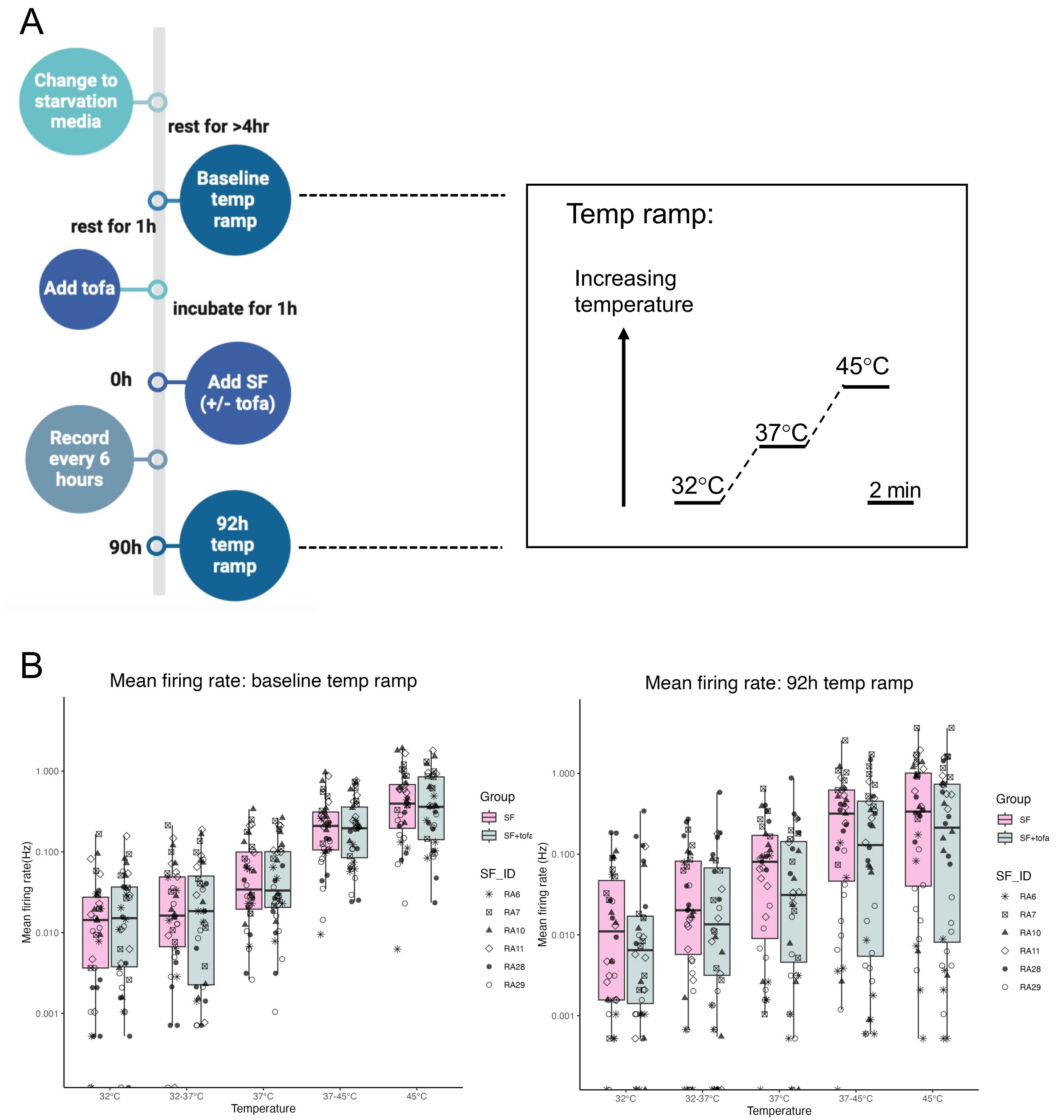

Supplementary Figure 9. MEA experiment procedure and analyses of iPSC-derived sensory neurons across temperature steps. **A.** Diagram showing the experimental procedure for RA SF (+/-tofa) experiments and the 32°C-45°C temperature ramp. During the temperature ramp, neuronal firing was recorded for 2 minutes at 32°C, 37°C and 45°C, and continuously during temperature increases from 32°C to 37°C and from 37°C to 45°C. **B.** Boxplots showing mean firing rate of Kute4 T52 and T55 neurons treated with 10% RA SF without or with JAKi at temperature steps from 32°C to 45°C. Figure legends represent the different RA SF and group. Neuronal age was D54 or D55 at baseline and D58 or D59 at 92 hours (92h). For both temperature ramps, there is a significant effect of temperature ($p < 0.0001$) calculated by repeated-measure ANOVA.

Supplementary tables

Supplementary Table 1. Reagents used in the study

Usage	Reagent	Company	Catalogue	Dilution/concentration
IPSC maintenance	Stemflex media	ThermoFisher	A3349401	NA
IPSC maintenance	Vitronectin	Stem cell technology	7180	40μL vitronectin in 1mL PBS
IPSC maintenance	Geltrex	ThermoFisher	A1569601	1:100 for IPSC, 1:50 for neurons
IPSC maintenance	Versene	ThermoFisher	15040066	NA
IPSC maintenance and replating	TrypLE	ThermoFisher	12604013	NA
Replating	Rho Kinase inhibitor Y-27632	Enzo	ALX-270-333-M005	10μM
Differentiation of IPSC into sensory neurons	Mouse Embryonic Fibroblast (MEF) Conditioned Media	Bio-techne	AR005	NA
Differentiation of IPSC into sensory neurons	Human FGF-2	Miltenyi	130-093-839	10 ng/mL
Differentiation of IPSC into sensory neurons	SB431542	Bio-techne	1614-10	10μM
Differentiation of IPSC into sensory neurons	LDN-193189	Bio-techne	6053-10	100nM
Differentiation of IPSC into sensory neurons	CHIR99021	Bio-techne	4423-10	3μM
Differentiation of IPSC into sensory neurons	SU5402	Merck	SML0443-5mg	10μM
Differentiation of IPSC into sensory neurons	DAPT	Bio-techne	2634-10	10μM
Components of Knockout DMEM media	Knockout DMEM basal	ThermoFisher	10829018	NA
Components of Knockout DMEM media	Knockout serum replacement	ThermoFisher	10829018	15%
Components of Knockout DMEM media	Glutamax	ThermoFisher	35050038	1%
Components of Knockout DMEM media	Nonessential amino acids	ThermoFisher	11140035	1%
Components of Knockout DMEM media	Beta-Mercaptoethanol	Gibco	31350-010	100 μM
Components of Knockout DMEM, N2 and stemflex media	Antibiotic/antimycotic	ThermoFisher	15240096	1%
Components of N2 media	Neurobasal medium	ThermoFisher	21103049	NA
Components of N2 media	B27 supplement	ThermoFisher	17504044	0.02

Supplementary Table 1. Reagents used in the study (continued)

Usage	Reagent	Company	Catalogue	Dilution/concentration
Components of N2 media	N2 supplement	ThermoFisher	17502048	0.01
Components of N2 media	Glutamax	ThermoFisher	35050038	0.01
Maintenance of IPSC-derived sensory neurons	Human β-NGF	Peprotech	450-01-100ug	25ng/mL (>day 39: 10ng/mL)
Maintenance of IPSC-derived sensory neurons	Human BDNF	Peprotech	450-02-100ug	25ng/mL (>day 39: 10ng/mL)
Maintenance of IPSC-derived sensory neurons	Human GDNF	Peprotech	450-10-100ug	25ng/mL (>day 39: 10ng/mL)
Maintenance of IPSC-derived sensory neurons	Human NT3	Peprotech	450-03-100ug	25ng/mL (>day 39: 10ng/mL)
Maintenance of IPSC-derived sensory neurons	Cytosine arabinoside (AraC)	SLS	C1768	1-3μM, depending on culture purity
Stimulation of IPSC-derived sensory neurons	IL-6	Proteintech	HZ-1019	100ng/mL
Stimulation of IPSC-derived sensory neurons	IL-6Ra	Proteintech	200-06RC-20ug	100ng/mL
Stimulation of IPSC-derived sensory neurons	LIF	Proteintech	HZ-1292	100ng/mL
Stimulation of IPSC-derived sensory neurons	IFN-alpha	Proteintech	HZ-1066-10UG (Lot: 121-02CD-08)	300U/mL (or 18ng/mL)
Stimulation of IPSC-derived sensory neurons	IFN-beta	Proteintech	HZ-1298-10UG (Lot: 119-07BJ-15)	300U/mL (or 13.5ng/mL)
Stimulation of IPSC-derived sensory neurons	IL-11	Proteintech	HZ-1333	100ng/mL
Stimulation of IPSC-derived sensory neurons	IL-11Ra	Biotechnne	8895-MR-050	100ng/mL
Stimulation of IPSC-derived sensory neurons	Tofacitinib	Cayman Chemical	11598	2μM or 10μM
Stimulation of IPSC-derived sensory neurons	Baricitinib	Cayman Chemical	16707	1μM
Stimulation of IPSC-derived sensory neurons	Upadacitinib	Cayman Chemical	29706	500nM
Western blot	RIPA buffer	ThermoFisher	89900	NA

Supplementary Table 1. Reagents used in the study (continued)

Usage	Reagent	Company	Catalogue	Dilution/concentration
Western blot	Halt™ Protease and Phosphatase Inhibitor Cocktail (100X)	ThermoFisher	78440	1:100
Western blot	BCA assay	ThermoFisher	23227	NA
Western blot	4x Laemmli Sample Buffer	Bio-Rad	1610747	NA
Western blot	beta-Mercaptoethanol	Gibco	31350-010	10%
Western blot	10x running buffer	ThermoFisher	LC26755	NA
Western blot	Trans-Blot Turbo RTA Mini 0.45 µm LF PVDF Transfer Kit, for 40 blots	Bio-Rad	1704274	NA
Western blot	Novex™ Tris-Glycine Mini Protein Gels, 8–16%, 1.0 mm, WedgeWell™ format: 15 wells	ThermoFisher	XP08165BOX	NA
Western blot	Skimmed milk	Sigma	70166	5% in TBS-T
Western blot	SuperSignal™ Pico Plus Chemiluminescent Substrate	ThermoFisher	34580	NA
Western blot	SuperSignal™ Femto Maximum Sensitivity Substrate	ThermoFisher	34094	NA
Western blot	SuperSignal™ West Atto Ultimate Sensitivity Substrate	ThermoFisher	A38554	NA
Western blot	Prestained protein marker	APExBIO	F4005	NA
Western blot	Bovine Serum Albumin (BSA)	Sigma	A7906	2%
Western blot	Stripping solution	Milliopore	504	NA
Western blot:primary antibody	pSTAT3 (Tyr705)	Cell signalling	mAb #9145T	1:2000
Western blot:primary antibody	STAT3	Cell signalling	mAb #12640	1:1000
Western blot: secondary antibody	Goat anti Rabbit-HRP	Abcam	Ab205718	1:5000
Immunocytochemistry: blocking	Donkey serum	VWR	S2170-500	1%, 5% or 10%
Immunocytochemistry: primary antibody	BRN3A (mouse)	Millipore	AB5945	1:100
Immunocytochemistry: primary antibody	PGP9.5 (rabbit)	Abcam	Ab108986	1:500
Immunocytochemistry: primary antibody	NF200 (Chicken)	Abcam	4680	1:500
Immunocytochemistry: primary antibody	pSTAT3 (Tyr705) (rabbit)	Cell signalling	mAb #9145T	1:100
Immunocytochemistry: secondary antibody	Donkey anti mouse-Alexa 488	Invitrogen	A21202	1:1000
Immunocytochemistry: secondary antibody	Donkey anti rabbit-Alexa 568	Invitrogen	A10042	1:1000
Immunocytochemistry: secondary antibody	Donkey anti chicken-Alexa 647	Invitrogen	A21449	1:1000
Immunocytochemistry: secondary antibody	Horse Anti-Rabbit IgG Antibody (H+L), Biotinylated	Vector Laboratory	BA-1100	1:500

Supplementary Table 1. Reagents used in the study (continued)

Usage	Reagent	Company	Catalogue	Dilution/concentration
Immunocytochemistry: tertiary antibody	Strapavidin 594	ThermoFisher	S11227	1:500
Immunocytochemistry: mounting media antibody	DAPI-containing mounting media	Invitrogen	00-4959-52	NA
Flow cytometry of IPSC cells	TRA-1-60 BV421	BD Biosciences	BD562711	1:100
Flow cytometry of IPSC cells	Live/dead-eFluor™ 780	ThermoFisher	65-0865-14	1:2000
RNA extraction	RNeasy Plus Kits for RNA Isolation	Qiagen	74134	NA
RNA quantification	Qubit™ dsDNA Quantification Assay Kits	ThermoFisher	Q32851	NA
cDNA conversion	SuperScript™ III Reverse Transcriptase	ThermoFisher	18080093	NA
qPCR	LightCycler® 480 SYBR Green I Master	Roche	4707516001	NA
Components of RPMI complete media	RPMI 1640 medium	Gibco	31870	NA
Components of RPMI and DMEM complete media	FCS, heat inactivated	Gibco	10500-064	10%
Components of RPMI and DMEM complete media	Penicillin-Streptomycin	Life Technologies	15070-063	1%
Components of RPMI and DMEM complete media	L-glutamine	Life Technologies	25030024	1% for RPMI; 2% for DMEM
Components of DMEM complete media	Dulbecco's Modified Eagle's Medium (DMEM) media	Sigma	D6546	NA
Fibroblast stimulation	IL-1beta	Peprotech	200-01B-10ug	10ng/mL
PBMC stimulation	Anti-human CD3 mAb clone OKT3	BioLegend	317326	1.25μg/mL
PBMC stimulation	Anti-human CD28 mAb clone CD28.2	BD Biosciences	555725	1μg/mL
Luminex	Luminex Discovery Assay Human Premixed Multi-analyte Kit	R&D Biotechnne	LXSAHM	NA
Luminex	Magneic plate separator	R&D Biotechnne	CN-0269-0	NA
Multi-electrode array (MEA)	Axion Cytoview 24-well plates	Axion	M384-tMEA-24W	NA
Multi-electrode array (MEA)	Poly(ethyleneimine) solution (PEI)	Sigma	P3143-500ML	0.1%
Multi-electrode array (MEA)	Borate buffer	Thermo Scientific	28341	Dilute to 1x

Supplementary Table 2. Primers used in this study. All were tested for their efficiency and specificity, and the correct product size was confirmed via gel electrophoresis.

Gene	Expressed by	Forward primer	Reverse primer
SCN9A	Sensory neurons	GTGCAGCACAGTTGATAACCC	AACCTCCGTACACAACCATCT
SST	Sensory neurons	CGGGGAAGCAGGAACTGG	GGCATCATTCTCCGTCTGGT
ATF3	Injured sensory neurons	GAAGAGCTGAGGTTTGCCATCCA	TGATTCCAGCGCAGAGGACATC
BDNF	Sensory neurons	TAACGGCGGCAGACAAAAAGA	TGCACTTGGTCTCGTAGAAGTAT
CSF1	Injured sensory neurons	TTAAGAAGGCATTTCTCCTG	CCTTGTCATGCTCTTCATAATC
FABP7	Non-neuronal cells (schwann cells)	GTCATCAGGACTCTCAGCACA	TCCAGGCTAACAACAGACTTACA
COL15A1	Non-neuronal cells (fibroblasts)	GCTTTCCAGCAACCCACATC	GCATGTTCAGAGCAGCCAAA
SOCS3	Many cell types	CAAGACCTTCAGCTCCAAGAG	TCACTGCGCTCCAGTAGAA
YWHAZ	House-keeping	TGTAGGAGCCCGTAGGTCATC	GTGAAGCATTGGGGATCAAGA
GAPDH	House-keeping	AGCCACATCGCTCAGACAC	GCCCAATACGACCAAATCC

Supplementary Table 3. Statistical tests performed in this study, including unit of analysis and observed effect size

Fig	Targets	Comparison	Unit of analysis	n	Observed effect size (d)	Statistical test	p-value	p-value cut-off post multiple comparison correction	Significant (*)
Fig.3C	pSTAT3	SF vs SF+tofa	Culture well: Kute4 JK T2	6	1.2	Paired nonparametric t-test	0.01256	0.05	*
Fig.4	IL-6	RA serum vs RA SF	Patient sample	12	0.98	Paired nonparametric t-test	0.00049	0.008	*
Fig.4	LIF	RA serum vs RA SF	Patient sample	12	1.08	Paired nonparametric t-test	0.00244	0.008	*
Fig.4	IL-11	RA serum vs RA SF	Patient sample	12	2.67	Paired nonparametric t-test	0.00049	0.008	*
Fig.4	OSM	RA serum vs RA SF	Patient sample	12	-0.32	Paired nonparametric t-test	0.46973	0.008	NS
Fig.4	IFN-alpha	RA serum vs RA SF	Patient sample	12	1.75	Paired nonparametric t-test	0.00592	0.008	*
Fig.4	IFN-beta	RA serum vs RA SF	Patient sample	12	1.66	Paired nonparametric t-test	0.00592	0.008	*
Supple. Fig3	IL-6	levels of mediator in RA SF vs neuronal pSTAT3/STAT3	Patient sample	10	0.79	Spearman's correlation	0.003	0.008	*
Supple. Fig3	LIF	levels of mediator in RA SF vs neuronal pSTAT3/STAT3	Patient sample	10	0.82	Spearman's correlation	0.002	0.008	*
Supple. Fig3	IL-11	levels of mediator in RA SF vs neuronal pSTAT3/STAT3	Patient sample	10	0.45	Spearman's correlation	0.09	0.008	NS
Supple. Fig3	OSM	levels of mediator in RA SF vs neuronal pSTAT3/STAT3	Patient sample	10	0.43	Spearman's correlation	0.11	0.008	NS
Supple. Fig3	IFN-alpha	levels of mediator in RA SF vs neuronal pSTAT3/STAT3	Patient sample	10	0.8	Spearman's correlation	0.003	0.008	*
Supple. Fig3	IFN-beta	levels of mediator in RA SF vs neuronal pSTAT3/STAT3	Patient sample	10	0.15	Spearman's correlation	0.34	0.008	NS
Fig.6B	SOCS3	IL-6+sIL-6R vs Ctrl	Culture well: Kute4 T35 UKB T9, T10	9	6.17	One-tailed nonparametric t-test	<0.0001	0.0125	*
Fig.6B	ATF3	IL-6+sIL-6R vs Ctrl	Culture well: Kute4 T35 UKB T9, T10	9	1.13	One-tailed nonparametric t-test	0.0094	0.0125	*
Fig.6B	BDNF	IL-6+sIL-6R vs Ctrl	Culture well: Kute4 T35 UKB T9, T10	9	0.91	One-tailed nonparametric t-test	0.017	0.0125	NS
Fig.6B	CSF1	IL-6+sIL-6R vs Ctrl	Culture well: Kute4 T35 UKB T9, T10	9	2.61	One-tailed nonparametric t-test	0.0001	0.0125	*
Fig.6C	SOCS3	IL-6+sIL-6R vs IL-6+sIL-6R+tofa	Culture well: Kute4 T40, UKB T12	6	3.17	One-tailed nonparametric t-test	0.001	0.0125	*
Fig.6C	ATF3	IL-6+sIL-6R vs IL-6+sIL-6R+tofa	Culture well: Kute4 T40, UKB T12	6	1.10	One-tailed nonparametric t-test	0.09	0.0125	NS
Fig.6C	BDNF	IL-6+sIL-6R vs IL-6+sIL-6R+tofa	Culture well: Kute4 T40, UKB T12	6	0.65	One-tailed nonparametric t-test	0.002	0.0125	*
Fig.6C	CSF1	IL-6+sIL-6R vs IL-6+sIL-6R+tofa	Culture well: Kute4 T40, UKB T12	6	2.62	One-tailed nonparametric t-test	0.004	0.0125	*
Fig.6C	SOCS3	LIF vs LIF+tofa	Culture well: Kute4 T40, UKB T12	6	2.59	One-tailed nonparametric t-test	0.001	0.0125	*
Fig.6C	ATF3	LIF vs LIF+tofa	Culture well: Kute4 T40, UKB T12	6	1.78	One-tailed nonparametric t-test	0.001	0.0125	*
Fig.6C	BDNF	LIF vs LIF+tofa	Culture well: Kute4 T40, UKB T12	6	1.29	One-tailed nonparametric t-test	0.001	0.0125	*
Fig.6C	CSF1	LIF vs LIF+tofa	Culture well: Kute4 T40, UKB T12	6	1.56	One-tailed nonparametric t-test	0.02	0.0125	NS
Fig.7A	Mean firing rate	LIF & temperature as independent variables	Culture well: Kute4 T40, UKB T14	14-16	NA	Repeated measures ANOVA	LIF (p=0.045), temperature(p=0.067)	NA	*
Fig. 7B	Mean firing rate	Group (SF, SF+tofa) & timepoints as independent variables	Culture well: Kute4 T52, Kute 4 T55	36	NA	Repeated measures ANOVA	Group (p=0.26), Timepoints (p<0.0001), Group x Timepoints (p=0. 029)	NA	*
Supple. 9B	Mean firing rate at Baseline temp ramp	Group (SF, SF+tofa) & Temperature as independent variables	Culture well: Kute4 T52, Kute 4 T55	36	NA	Repeated measures ANOVA	Group (p=0.83), temperature(p<0.0001), Temperature x Group (p=0.97)	NA	NS
Supple. 9B	Mean firing rate at 92h temp ramp	Group (SF, SF+tofa) & Temperature as independent variables	Culture well: Kute4 T52, Kute 4 T55	36	NA	Repeated measures ANOVA	Group (p=0.45), temperature(p<0.0001), Temperature x Group (p=0.76)	NA	NS

Observed effect size was calculated as Cohen‘s $d = (M_2 - M_1) / \sqrt{((SD_1^2 + SD_2^2) / 2)}$. In experiments where multiple statistical tests were performed, Bonferroni correction was performed. When the observed p value was lower than the new p-value cut-off, * was added. NS: Not significant. Wilcoxon signed-rank tests were used for paired nonparametric t tests and Mann Whitney U test for the one-tailed, independent nonparametric t tests.

Supplementary Table 4. Luminex assay information

	HC serum (pg/mL): Mean (SD)	RA serum (pg/mL): Mean (SD)	RA SF (pg/mL): Mean (SD)	Standard1 (pg/mL)	Standard6 (pg/mL)	Sensitivity (pg/mL)	Standard curve-fit R^2
**IL-6**	130.0 (112.0)	179.4 (382.0)	17734.4 (25368.1)	79000	325.1	170.0	0.997
**IFN-beta**	1.9 (0.0)	1.9 (0.0)	21.0 (16.1)	6880	28.3	1.0	0.903
**IL-6R alpha**	36477.9 (5306.9)	36286.4 (11374.5)	36909.2 (12379.4)	116380	478.9	1.2	0.998
**Oncostatin M/OSM**	7952.3 (581.6)	7740.9 (839.4)	7324.8 (1653.3)	186460	767.3	88.6	0.962
**LIF**	12.2 (8.8)	15.1 (10.1)	57.1 (54.3)	11780	48.5	18.6	0.996
**IFN-alpha**	1.3 (0.0)	1.9 (2.2)	62.0 (48.5)	5400	22.2	0.5	0.999
**IL-11**	182.1 (0.0)	478.3 (707.1)	3310.5 (1324.0)	213600	879	49.4	0.926

HC serum, RA serum and RA SF were diluted 1:2 and 1:100 and run on the same Luminex plate. 1:100 dilution wells were chosen to calculate IL-6 concentration and 1:2 wells were chosen to calculate the concentration for the rest of the analytes, based on which dilution had most datapoints fall within the standard curve (STD1-STD6). The standard curve was derived using a five parameter logistic (5-PL) curve-fit, calculated by xPonent software upon running the plate on a FLEXMAP 3D machine according to manufacturer's instructions. Standard 1 (top standard), Standard 6 (bottom standard) and sensitivity have taken the corresponding dilution factors into account. Demographicand clinical details of the samples used are provided in **Table 1**. Sensitivity information was obtained on the R&D website when designing the panel: biotechne.com/l/rl/t9MRQ6Nj.

Supplementary methods

1. Bioinformatic analyses

We derived information from five previously published RNA sequencing datasets: two single-nuclei sequencing of post-mortem human sensory neurons, GSE168243 and GSE201586 (1, 2), two bulk RNA sequencing datasets of IPSC-derived sensory neurons, one of which was of neurons we grew in-house (GSE268585) and a compilation dataset (3). For the single-nuclei RNA sequencing datasets, we generated pseudobulk profiles for each biological replicate and plotted the expression of our genes of interest.

2. Fibroblast and PBMC/SFMC culture

Cryopreserved RA synovial fibroblasts were thawed and cultured at 37°C with 5% CO2 in DMEM complete medium, containing 10% FCS, 1% penicillin/streptomycin, 2% L-glutamine and 1µg/mL Amphotericin B/Fungizone. Three RA patient-derived fibroblast lines at passage 5-6 were used in this study. Fibroblasts were seeded in 12-well plates (60,000 cells/well) the night before the experiment and subsequently stimulated with or without IL-1beta for 24 hours.

Cryopreserved healthy donor and RA patient PBMC and/or SFMC were thawed and cultured in 24-well plates (1 million cells/well) in RPMI complete medium only or with plate-bound anti-CD3/soluble anti-CD28 mAbs for 3 days at 37°C with 5% CO2. Complete RPMI medium was composed of RPMI 1640 medium containing 10% FCS, 1% Penicillin/Streptomycin and 1% L-glutamine. Conditioned media were harvested by spinning down the cells at 5900g for 3.5 minutes. Supernatants were removed and stored at -80˚C until Luminex assessment.

3. Sensitivity analyses

For Luminex experiments, using n=12 patient samples, we conducted paired non-parametric t-tests to compare the levels of our target cytokines in RA serum and SF. Since we measured six cytokines, the p-value cut-off was lowered to 0.008 using Bonferroni correction. Sensitivity analysis suggests this design will provide an 80% chance to detect effects size of d=1.26 or larger. Using n=10 patient samples, we also correlated the levels of mediators in SF with the levels of neuronal pSTAT3/STAT3 they induced as measured by Western blot. p=0.008 was used as a cut-off, with sensitivity analysis indicating that this will provide an 80% chance of seeing correlations of rho=0.83 or larger.

For gene expression analyses, using n=6-9 culture wells, we conducted one-tailed non-parametric t-tests to see whether IL-6+sIL-6R or LIF increased the expression of *SOCS3*, *ATF3*, *BDNF* and *CSF1*. Since four genes were tested, the p-value cut-off was lowered to 0.0125 using Bonferroni correction. Sensitivity analyses suggest this design has an 80% chance to detect effects of d=1.62-2.11.

References:

1. Jung M, Dourado M, Maksymetz J, Jacobson A, Laufer BI, Baca M, et al. Cross-species transcriptomic atlas of dorsal root ganglia reveals species-specific programs for sensory function. Nature Communications. 2023;14(1):366.
2. Nguyen MQ, von Buchholtz LJ, Reker AN, Ryba NJ, Davidson S. Single-nucleus transcriptomic analysis of human dorsal root ganglion neurons. Elife. 2021;10.
3. An integrated encyclopedia of DNA elements in the human genome. Nature. 2012;489(7414):57-74.
