# Supplementary material for: Rheumatoid arthritis synovial fluid induces JAK-dependent intracellular activation of human sensory neurons
Source: JCI Insight. 2025 May 15;10(12):e186646. doi: 10.1172/jci.insight.186646 (PMC12220938; doi:10.1172/jci.insight.186646)
Supplement: Unedited blot and gel images [file jciinsight-10-186646-s128.pdf]

All Western blots used in manuscript

Yuening Li

2024.8

Note: Only pSTAT3 and STAT3 blots were used in the manuscript.
The ECL kit to image the blots are indicated on the lower right corners.
The relevant lanes used in the manuscript are indicated in each slide and marked by:

Figure 3A

Used in manuscript: RA1, ..., RA10, No stim

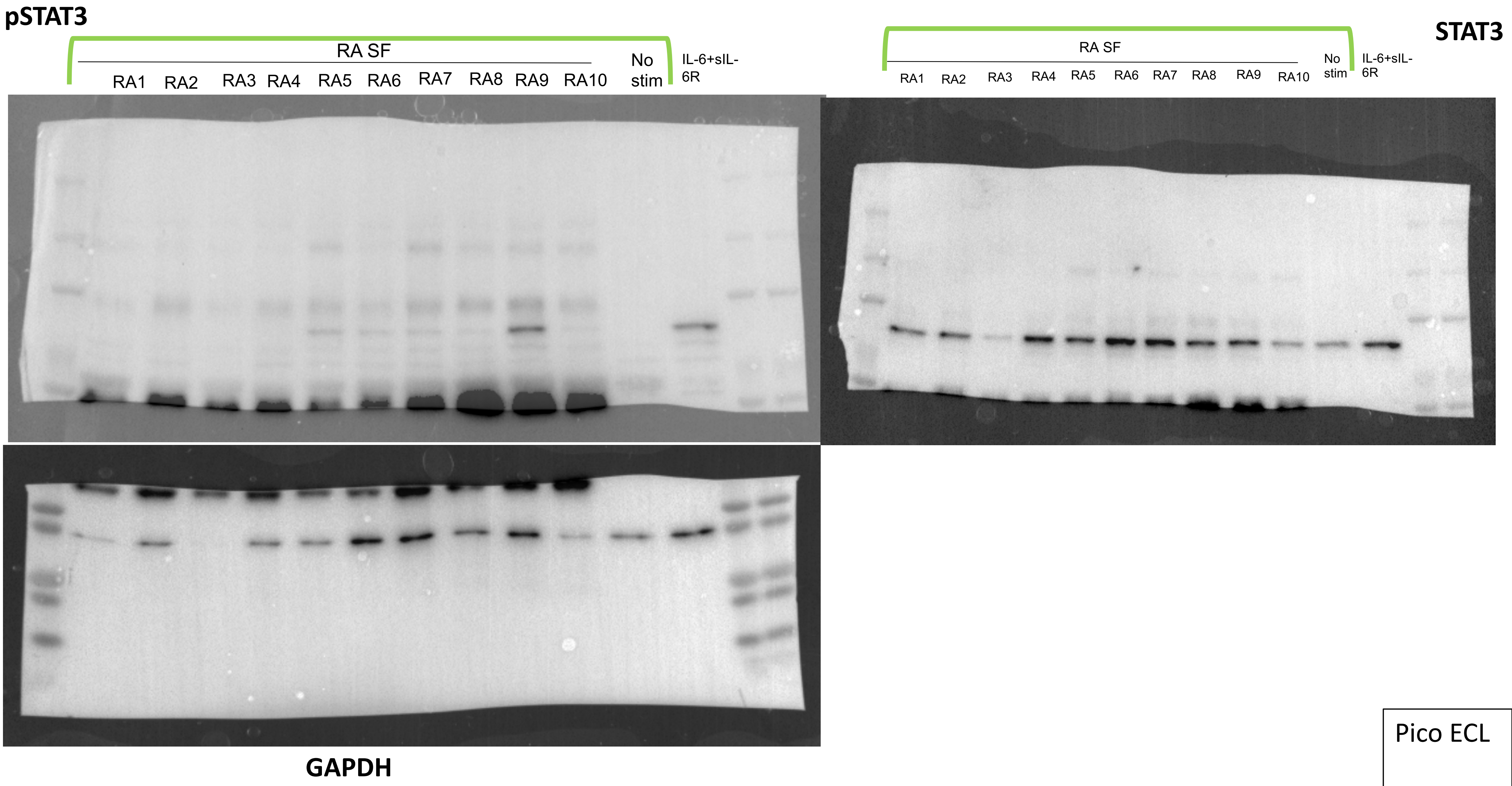

Figure 3B

Used in manuscript: RA10, RA10 tofa, RA11, RA11 tofa, RA8, RA8, tofa

pSTAT3

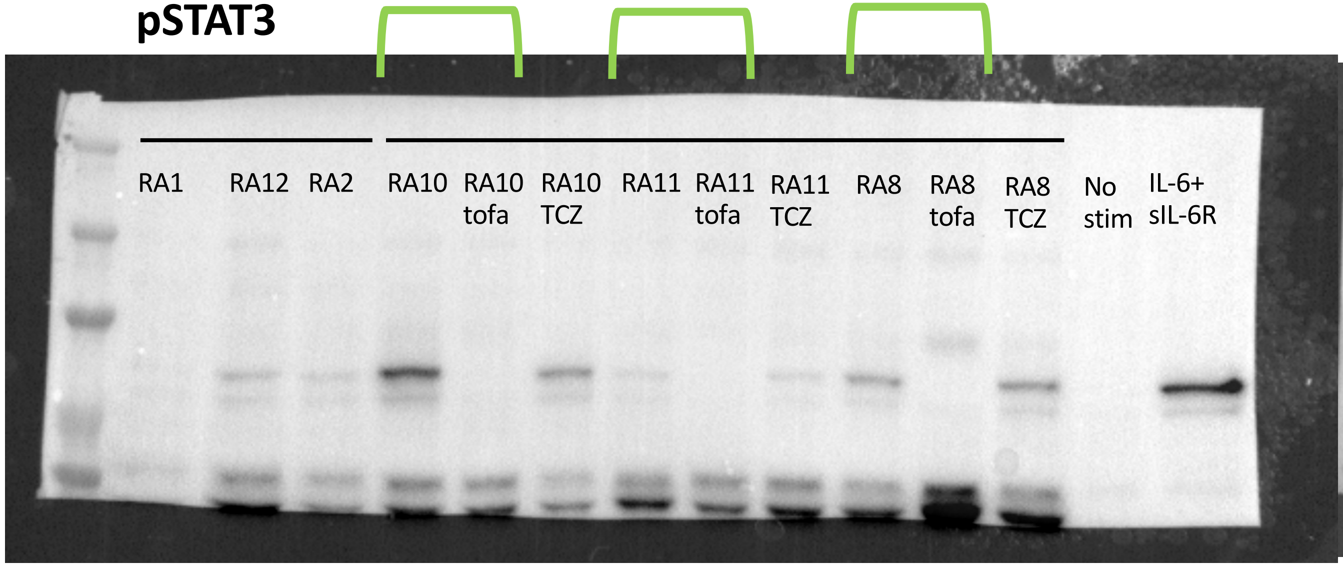

STAT3

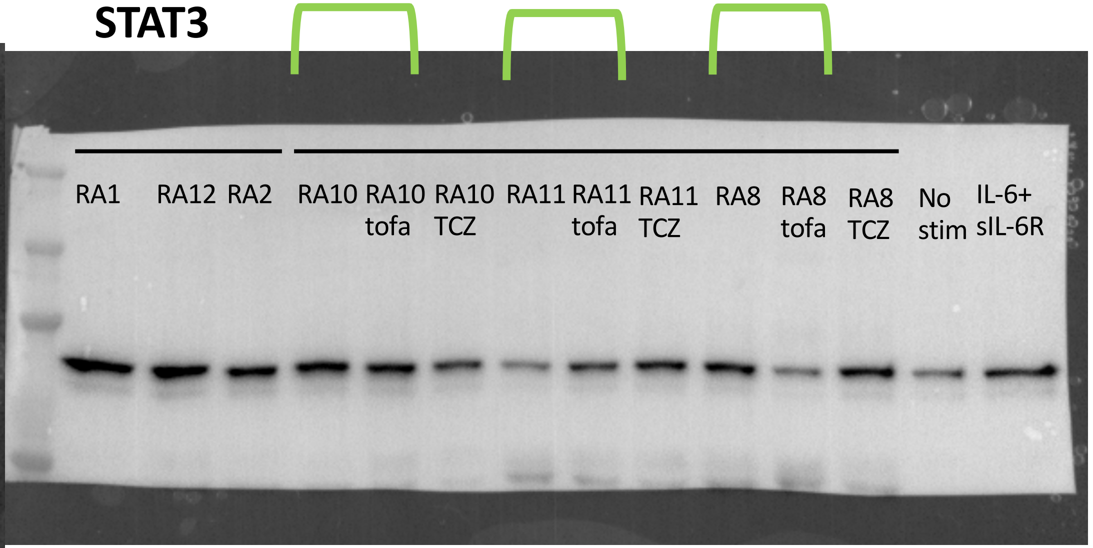

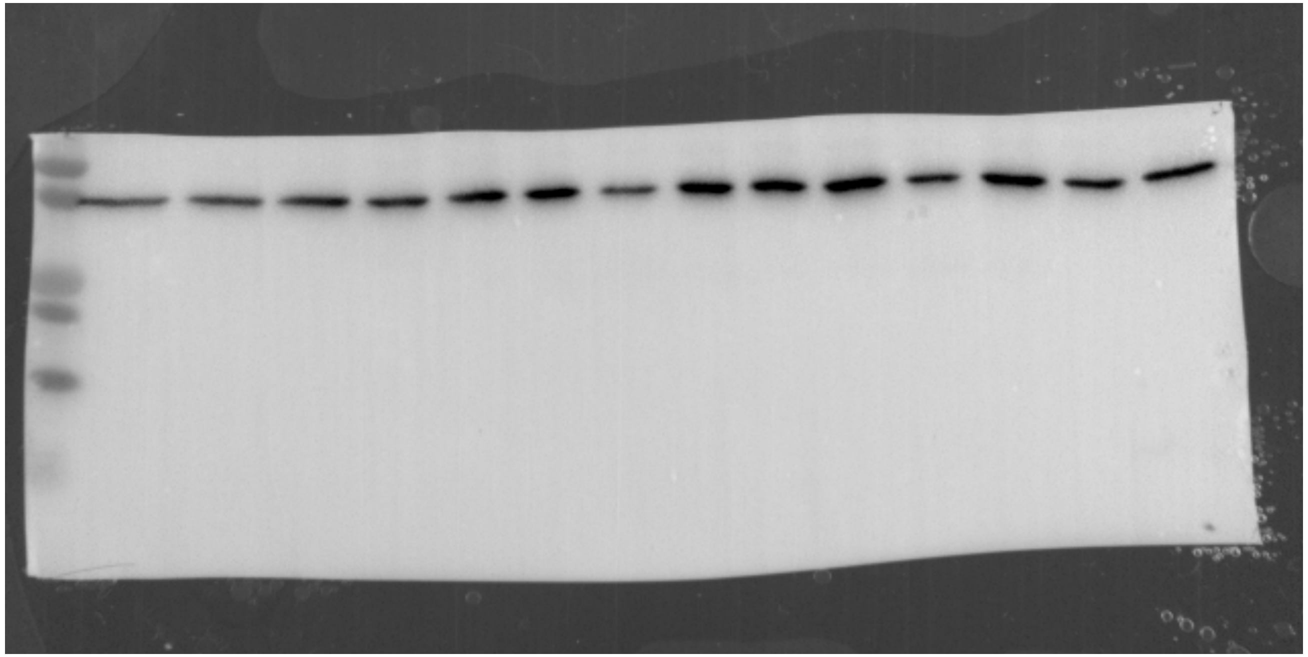

GAPDH

Tofa: tofacitinib
TCZ: tocilizumab

Pico ECL

Figure 5A

Used in manuscript: all conditions

pSTAT3

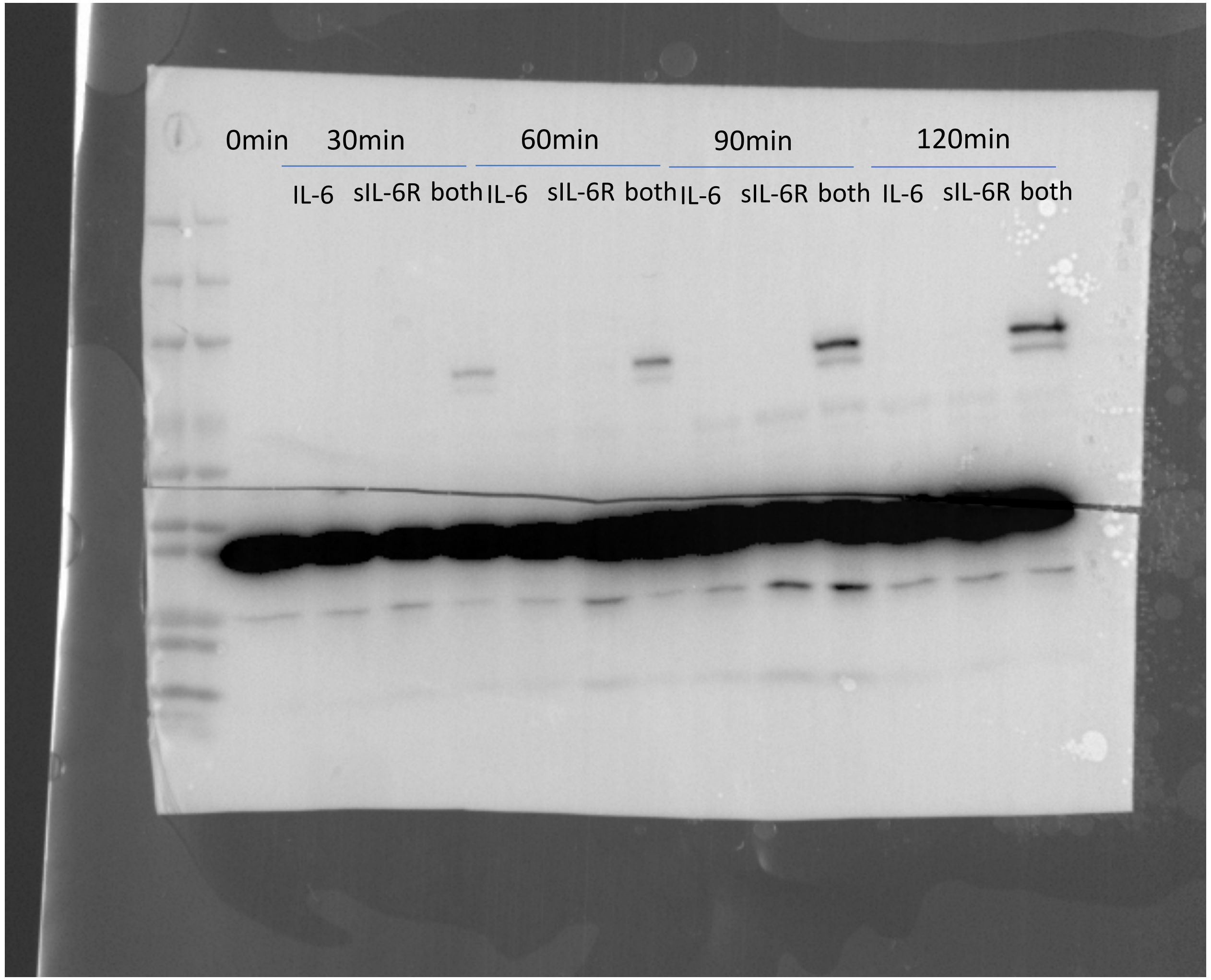

GAPDH

STAT3

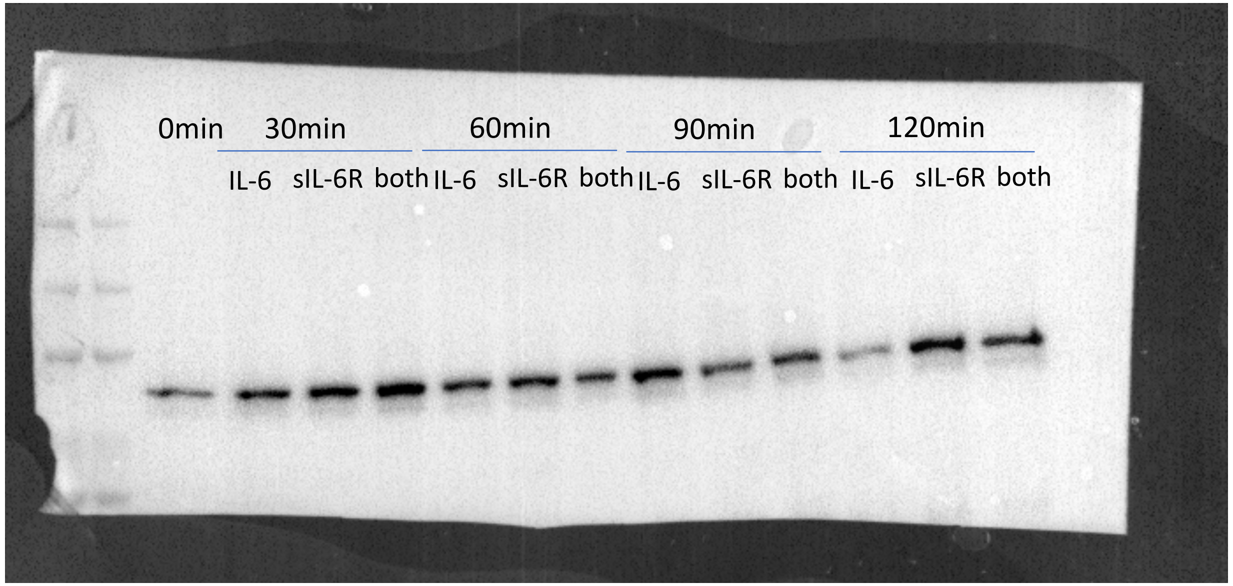

Pico ECL

Figure 5B

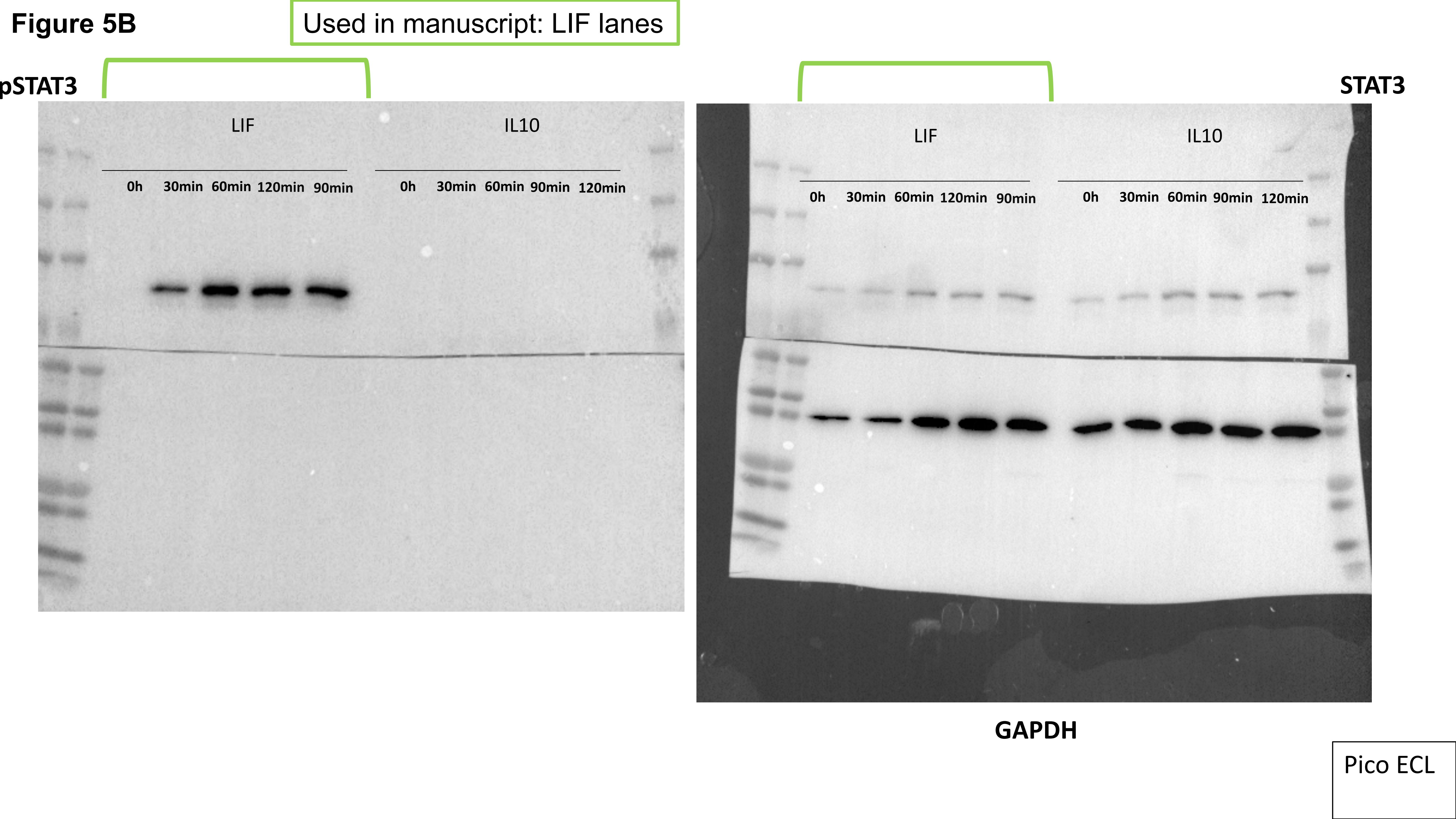
Used in manuscript: LIF lanes
pSTAT3
LIF
IL10
0h
30min
60min
120min
90min
0h
30min
60min
90min
120min
STAT3
LIF
IL10
0h
30min
60min
120min
90min
0h
30min
60min
90min
120min
GAPDH
Pico ECL

Figure 5C

Used in manuscript: IFNα & IFNβ conditions

pSTAT3

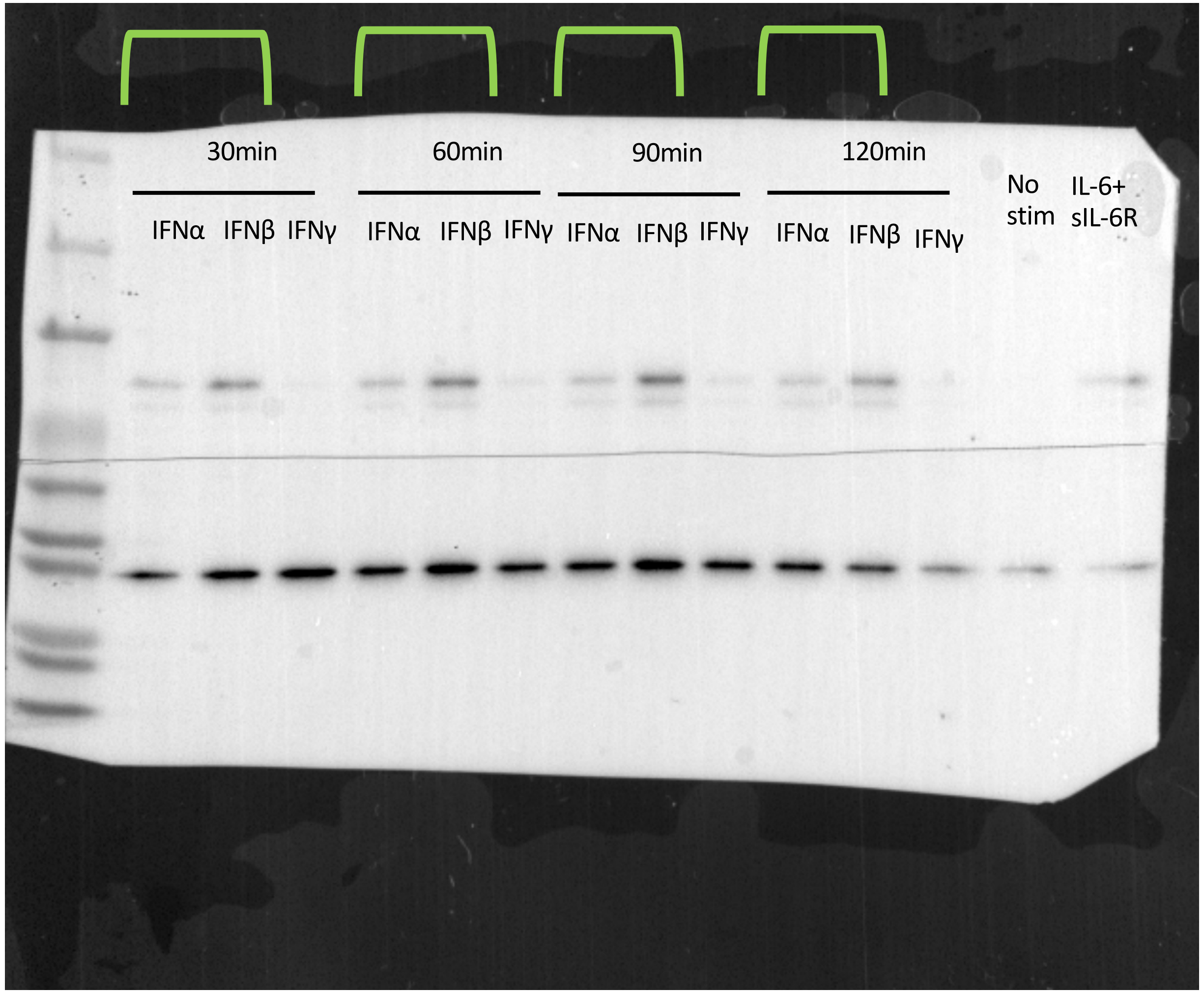

GAPDH

STAT3

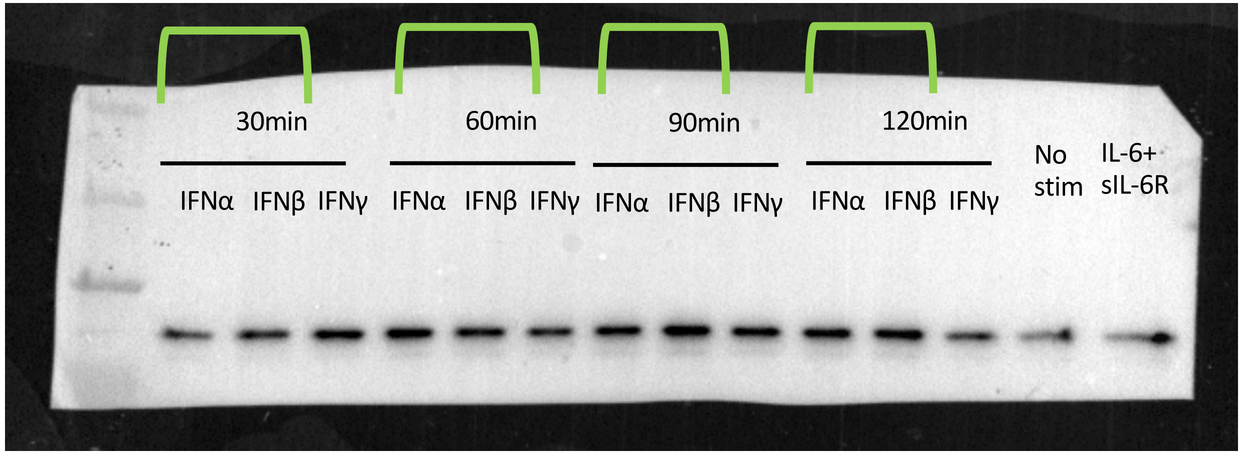

pSTAT3: Atto ECL
STAT3: Femto ECL

Figure 5D

Used in manuscript: 30min to 120min conditions

pSTAT3

STAT3

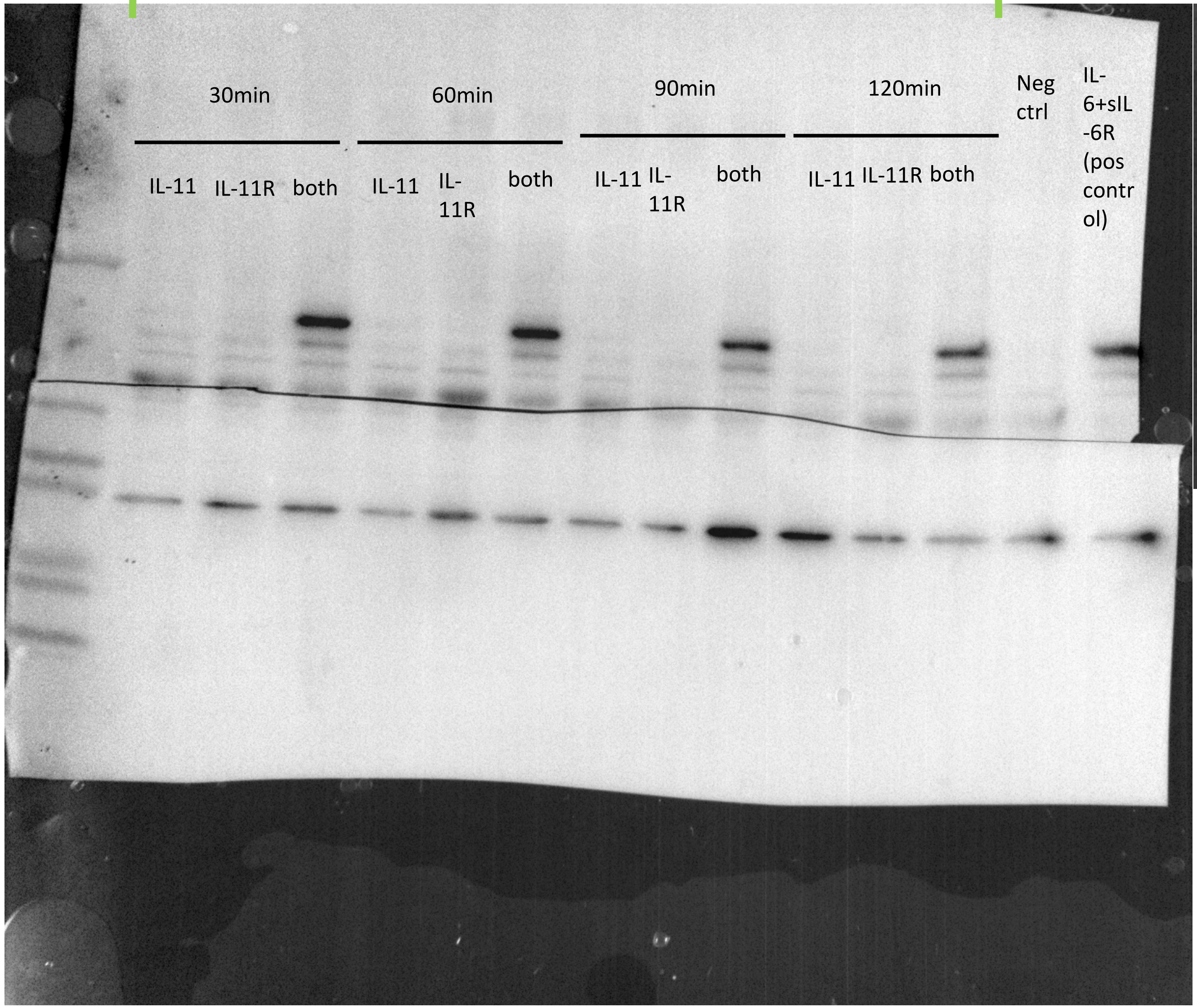

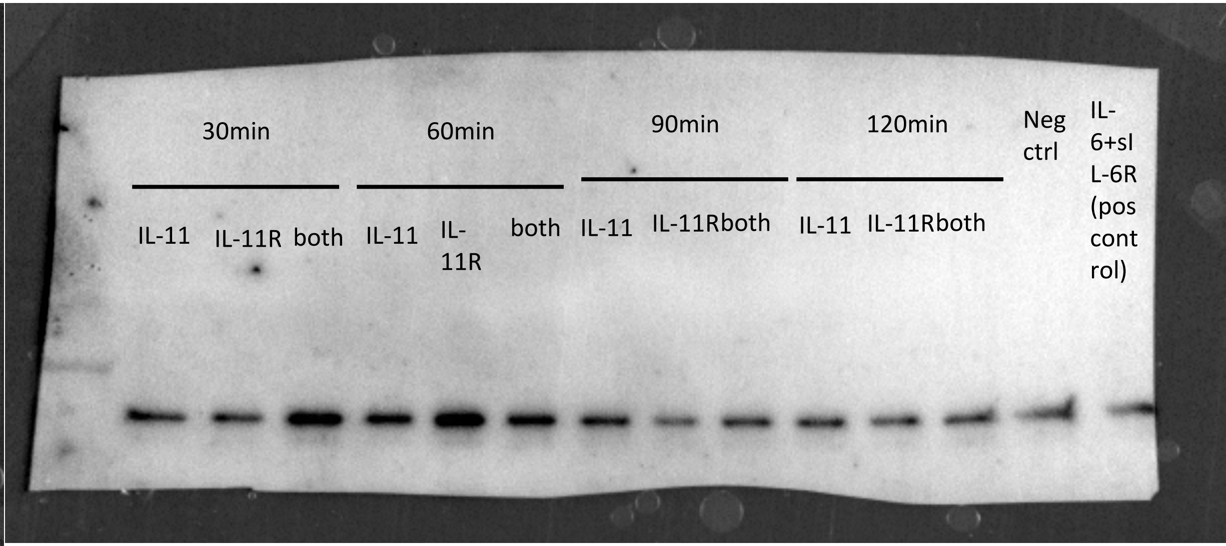

GAPDH

pSTAT3: Atto ECL
STAT3: Femto ECL

Figure 5E

Used in manuscript: No stim, IL-6+sIL-6R, LIF conditions

pSTAT3

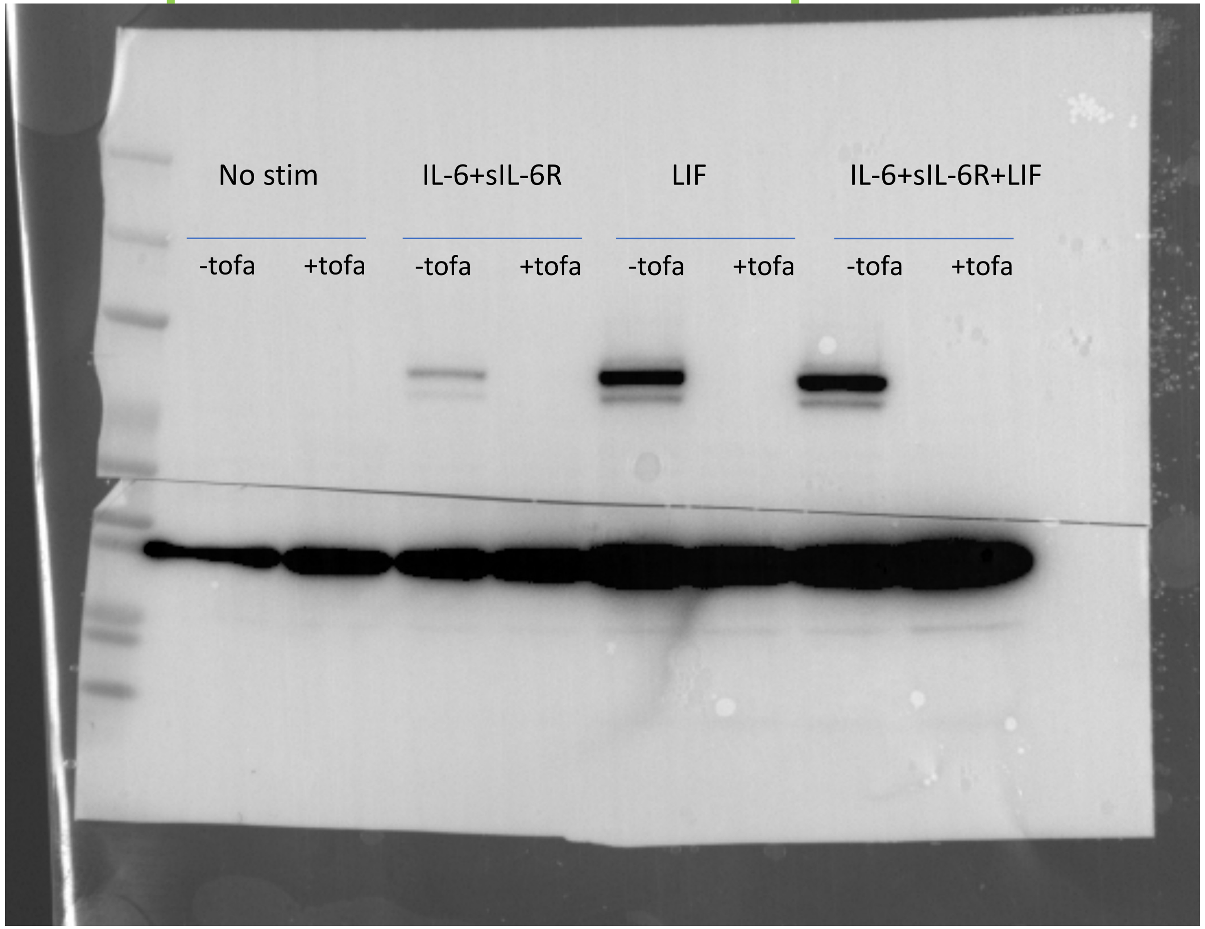

STAT3

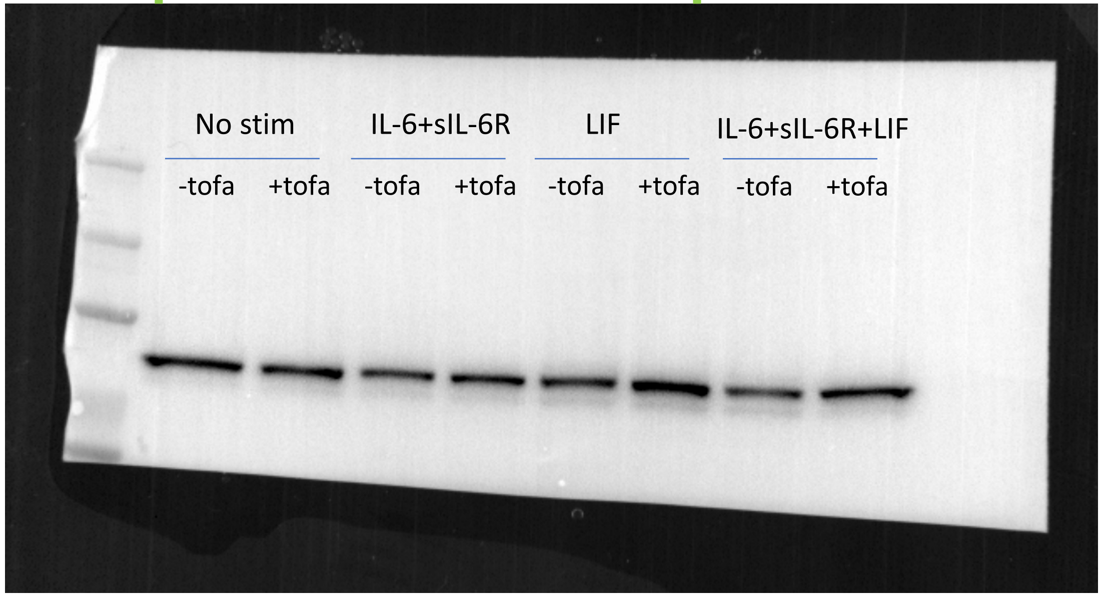

GAPDH

Pico ECL

Supplementary Fig. 2A

Used in manuscript: all but IL-6+sIL-6R condition

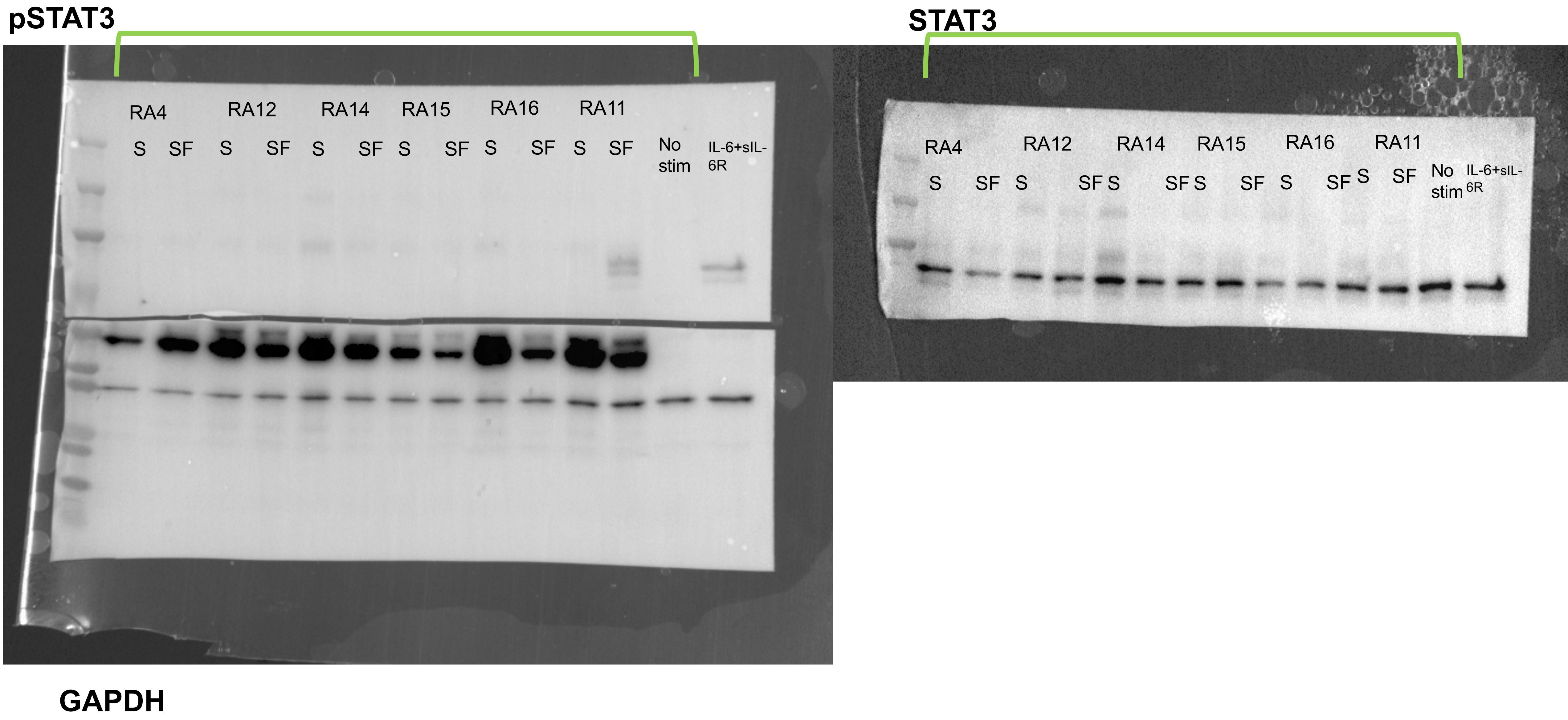

S: serum

Pico ECL

Supplementary Figure 6A

Used in manuscript: all but the last two lanes

pSTAT3

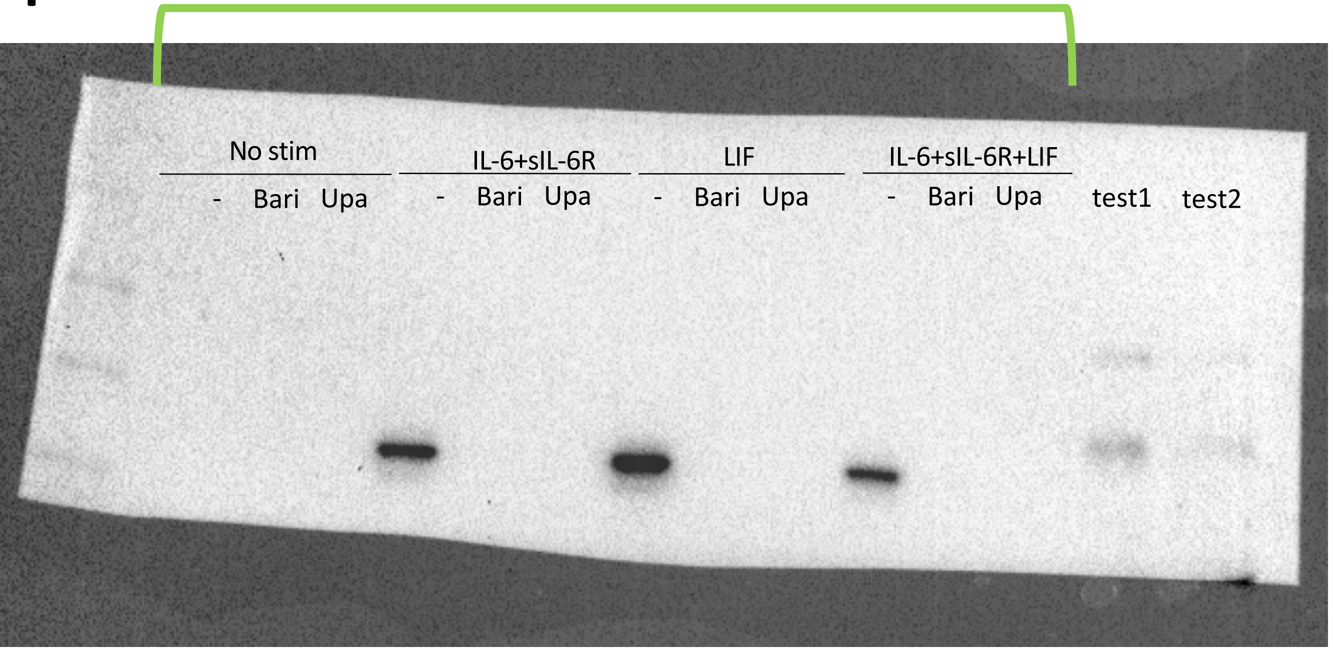

STAT3

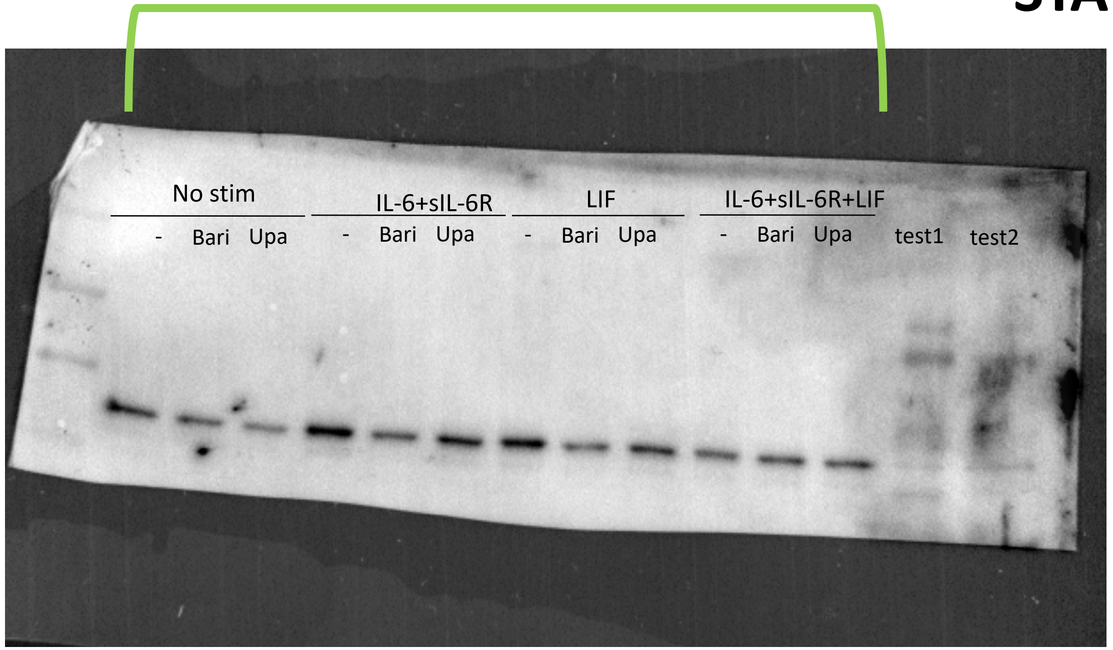

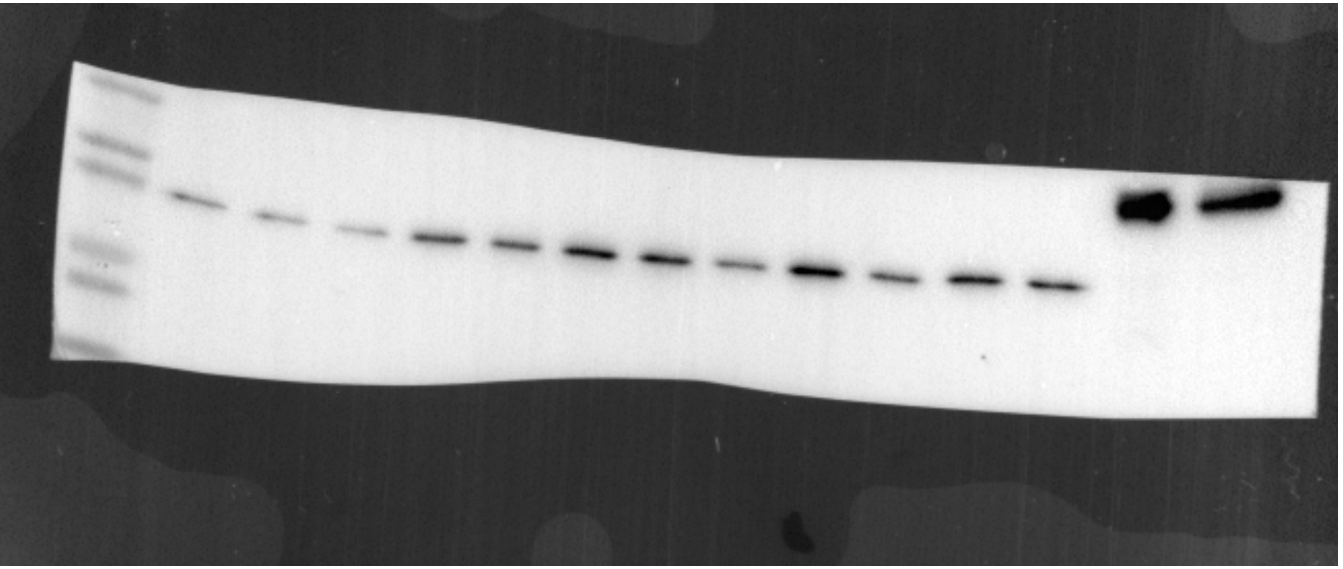

GAPDH

pSTAT3: pico ECL
STAT3: atto ECL
